# Antidepressant use and risk of adverse outcomes in people aged 20–64 years: cohort study using a primary care database

**DOI:** 10.1186/s12916-018-1022-x

**Published:** 2018-03-08

**Authors:** Carol Coupland, Trevor Hill, Richard Morriss, Michael Moore, Antony Arthur, Julia Hippisley-Cox

**Affiliations:** 1 Division of Primary Care, University of Nottingham, 13th floor, Tower Building, University Park, Nottingham, NG7 2RD UK; 2 Institute of Mental Health, Jubilee Campus, Wollaton Road, Nottingham, NG8 1BB UK; 3 University of Southampton Medical School, Primary Care and Population Sciences, Aldermoor Health Centre, Aldermoor Close, Southampton, SO16 5ST UK; 4 School of Nursing Sciences, Faculty of Medicine and Health Sciences, Edith Cavell Building, University of East Anglia, Norwich Research Park, Norwich, NR4 7TJ UK

**Keywords:** Antidepressants, Depression, Fracture, Falls, Gastrointestinal bleed, Mortality, Adverse effects

## Abstract

**Background:**

Antidepressants are one of the most commonly prescribed medications in young and middle-aged adults, but there is relatively little information on their safety across a range of adverse outcomes in this age group. This study aimed to assess associations between antidepressant treatment and several adverse outcomes in people aged 20–64 years diagnosed with depression.

**Methods:**

We conducted a cohort study in 238,963 patients aged 20–64 years registered with practices across the UK contributing to the QResearch primary care database. Only patients with a first diagnosis of depression were included. Outcomes were falls, fractures, upper gastrointestinal bleed, road traffic accidents, adverse drug reactions and all-cause mortality recorded during follow-up. Cox proportional hazards models were used to estimate hazard ratios associated with antidepressant exposure adjusting for potential confounding variables.

**Results:**

During 5 years of follow-up, 4651 patients had experienced a fall, 4796 had fractures, 1066 had upper gastrointestinal bleeds, 3690 had road traffic accidents, 1058 had experienced adverse drug reactions, and 3181 patients died. Fracture rates were significantly increased for selective serotonin reuptake inhibitors (adjusted hazard ratio 1.30, 95% CI 1.21–1.39) and other antidepressants (1.28, 1.11–1.48) compared with periods when antidepressants were not used. All antidepressant drug classes were associated with significantly increased rates of falls. Rates of adverse drug reactions were significantly higher for tricyclic and related antidepressants (1.54, 1.25–1.88) and other antidepressants (1.61, 1.22–2.12) compared with selective serotonin reuptake inhibitors. Trazodone was associated with a significantly increased risk of upper gastrointestinal bleed. All-cause mortality rates were significantly higher for tricyclic and related antidepressants (1.39, 1.22–1.59) and other antidepressants (1.26, 1.08–1.47) than for selective serotonin reuptake inhibitors over 5 years but not 1 year, and were significantly reduced after 85 or more days of treatment with selective serotonin reuptake inhibitors. Mirtazapine was associated with significantly increased mortality rates over 1 and 5 years of follow-up.

**Conclusions:**

Selective serotonin reuptake inhibitors had higher rates of fracture than tricyclic and related antidepressants but lower mortality and adverse drug reaction rates than the other antidepressant drug classes. The association between mirtazapine and increased mortality merits further investigation. These risks should be carefully considered and balanced against potential benefits for individual patients when the decision to prescribe an antidepressant is made.

**Electronic supplementary material:**

The online version of this article (10.1186/s12916-018-1022-x) contains supplementary material, which is available to authorized users.

## Background

Depression is a serious condition, common in adults of all ages worldwide [1, 2]. It is frequently treated with antidepressant drugs, with many countries reporting substantial increases in the prescribing rates of these drugs in recent decades [3–5]. Reports from the US, Canada and UK have shown that antidepressants are one of the most commonly prescribed types of medication in young and middle-aged adults [6–9], taken by 7% of adults aged 18–39 years and by 14% of adults aged 40–59 in the US [9]. Several different types of antidepressant drug are available, with broadly equal efficacy, although there is ongoing debate over their effectiveness compared to placebo [10–12]. Therefore, the choice of an antidepressant largely depends on the consideration of potential adverse effects. Guidelines recommend that selective serotonin reuptake inhibitors should generally be considered as the first-line treatment for depression [13].

Despite the widespread use of antidepressants, there is relatively little information on their safety across a range of serious adverse outcomes in young and middle-aged adults. Adverse effects have been evaluated in randomised controlled trials, but these trials are usually in select groups and are relatively small and short term, therefore lacking the power to detect rare but serious adverse effects. Observational studies have shown associations between the use of selective serotonin reuptake inhibitors and increased risks of fractures and falls [14, 15], but these studies have either been carried out only in older people or have been dominated by outcomes occurring in older people where event rates are higher. There is some indication that antidepressants may impair the ability to drive in older people, but the evidence in younger drivers is equivocal [16]. Similarly studies have found increased risks of gastrointestinal bleeds [17], adverse drug reactions [18] and all-cause mortality [19] associated with antidepressant use, but there is a lack of evidence in young and middle-aged adults, where patterns of risk may differ compared with older people due to greater levels of comorbidity, interactions with other prescribed medications, and increased susceptibility to adverse effects in older populations [20, 21].

Given the lack of evidence regarding antidepressant safety in a younger population despite the large numbers of prescriptions issued to this group for increasingly long durations, we performed a large cohort study in people aged 20–64 years in order to investigate the associations between different antidepressant drugs and the risks of several potential adverse outcomes. We aimed to provide a comprehensive assessment of risks by drug class, and for the most commonly prescribed individual antidepressant drugs.

## Methods

The cohort study was designed to assess associations between antidepressant treatment and several different adverse outcomes, including falls, fractures, upper gastrointestinal bleed, road traffic accident, adverse drug reaction, and all-cause mortality. Findings for suicide, self-harm, epilepsy and cardiovascular outcomes have been previously reported [22–24]. Full details of the study design and methods can be found in the study protocol [25].

### Study cohort

A large primary care database (QResearch, version 34) was used to select the study cohort. At the time of the study the QResearch database included health records of over 12 million patients from more than 600 general practices across the United Kingdom which record data using the Egton Medical Information Systems (EMIS) medical records computer system. The information recorded includes patient characteristics, clinical diagnoses, symptoms and prescribed medications.

The study cohort included patients aged 20–64 years with a first recorded diagnosis of depression between January 1, 2000, until July 31, 2011. Diagnostic Read codes, which are the standard clinical codes used in general practice in the United Kingdom, were used to identify patients with a diagnosis of depression, using codes employed in previous studies [26–28]. Patients were only eligible for inclusion if their diagnosis of depression occurred at least 12 months after their registration with a study practice and the installation date of their practice’s EMIS computer system to ensure it was a new diagnosis and not a retrospective recording of a previous diagnosis.

Patients were excluded if they had a previous recorded diagnosis of depression, or if they had received prescriptions for an antidepressant either before the study start date (January 1, 2000), before their registration date, before the age of 20, or more than 36 months before their first recorded diagnosis of depression. We excluded patients with a previous diagnosis of depression or prescriptions for antidepressants more than 36 months prior to diagnosis so that antidepressant prescribing during follow-up would not be influenced by any prior experiences or preferences that would be difficult to account for in the analyses. Where patients were prescribed antidepressants within the 36 months before their recorded diagnosis of depression we assumed that these were being prescribed for depression. Patients were also excluded if they were temporary residents due to lack of follow-up data or if they had a diagnosis of schizophrenia, bipolar disorder or another type of psychosis, or had been prescribed lithium or antimanic drugs to reduce indication bias.

Each patient’s study entry date was defined as the date of the first recorded diagnosis of depression, or the date of the first prescription for an antidepressant if that was earlier. Patients were then followed up until the earliest date of leaving the practice, death or end of the follow-up period (August 1, 2012).

### Outcomes

The outcomes for these analyses were falls, fractures (including vertebral, rib, pelvis, upper limb, lower limb, distal radius, hip and skull fractures), upper gastrointestinal bleed, road traffic accidents, adverse drug reactions and all-cause mortality. Patients with these outcomes were identified if they were recorded either on the patients’ general practice record using the relevant Read codes or on their linked Office of National Statistics cause of death record using International Classification of Diseases diagnostic codes, employing codes similar to those used in previous studies [29–31]. The adverse drug reactions outcome included specific codes for adverse reactions to antidepressants, codes for adverse drug reactions where the drug was not specified and codes for bullous eruption. Patients with a previous diagnosis of an outcome were excluded from the analysis of the respective outcome.

Antidepressant poisoning and sudden death were two further pre-specified outcomes [25], but numbers of patients with these outcomes recorded were too small for further analysis.

### Exposures

Information was extracted from all prescriptions for antidepressants issued during follow-up. We calculated the duration of each prescription in days by dividing the number of tablets prescribed by the number of tablets to be taken each day. If the information on tablets per day was missing or not sufficiently detailed (< 5% of total prescriptions) we estimated the duration of the prescription based on the number of tablets prescribed, as in our previous study [29]. Antidepressant drugs were grouped according to the four main classes in the British National Formulary: tricyclic and related antidepressants, selective serotonin reuptake inhibitors, monoamine oxidase inhibitors and other antidepressants. If different antidepressant drugs were prescribed on the same date these were classified as combined prescriptions. Patients were classified as continually exposed to an antidepressant during periods where there were no gaps of more than 90 days between the end of one prescription and the start of the next. Patients were also classified as exposed for the first 90 days after the estimated date of stopping an antidepressant in order to account for any delays in starting the prescription or accumulation of tablets as well as to attribute the outcomes occurring during withdrawal periods to the antidepressant.

The daily dose of each prescription was calculated by multiplying the number of tablets to be taken each day by the dose of each tablet, and then converted to a defined daily dose using values assigned by the World Health Organization’s Collaborating Centre for Drug Statistics Methodology (www.whocc.no/atc_ddd_index). The 11 most commonly prescribed individual antidepressant drugs were also assessed separately, as in our previous study [26].

### Confounding variables

Confounders were variables considered to be potential risk factors for the outcomes or associated with the likelihood of receiving a particular antidepressant treatment, based on our previous study of antidepressants in people aged 65 or over [26]. These were age at study entry; sex; year of diagnosis of depression; severity of index diagnosis of depression (categorised as mild, moderate or severe, using codes published by Martinez et al. [27] and some further classification of additional codes included in this study by a member of the study team); deprivation (Townsend deprivation score corresponding to the patients postcode, in fifths); smoking status (non-smoker, ex-smoker, light smoker: 1–9 cigarettes/day, moderate smoker: 10–19 cigarettes/day, heavy smoker: ≥ 20 cigarettes/day, not recorded); alcohol intake (none, trivial: < 1 unit/day, light: 1–2 units/day, medium: 3–6 units/day, heavy: 7–9 units/day, very heavy: > 9 units/day, not recorded); ethnic group (categorised as either white/not recorded or non-white (Indian, Pakistani, Bangladeshi, other Asian, black African, black Caribbean, Chinese, other including mixed)); comorbidities at baseline (binary variables for each of coronary heart disease, stroke/transient ischaemic attack, diabetes, hypertension, cancer, epilepsy/seizures, hypothyroidism, osteoarthritis, rheumatoid arthritis, asthma/chronic obstructive airways disease, osteoporosis, liver disease, renal disease, obsessive-compulsive disorder); and use of other drugs at baseline (binary variables for each of antihypertensive drugs, aspirin, statins, anticoagulants, non-steroidal anti-inflammatory drugs, anticonvulsants, hypnotics/anxiolytics, anti-psychotics, bisphosphonates, oral contraceptives, hormone replacement therapy). In addition, a record of falls at baseline was included as a confounding variable for the fracture outcome.

### Statistical analysis

Cox’s proportional hazards model was used to estimate associations between each of the outcomes and antidepressant drug exposure, using robust standard errors to allow for clustering of patients within practices and excluding patients from the analysis if they had the outcome recorded at baseline. The main analyses were based on the first 5 years of follow-up after study entry. We selected 5 years of follow-up for the main analyses as this includes periods of long-term treatment and allows more events to accrue to increase the power of the study compared with a shorter period. Patients prescribed monoamine oxidase inhibitors at any time were excluded from the analyses due to small numbers.

The analysis calculated unadjusted and adjusted hazard ratios (HRs) by antidepressant class treated as a time-varying exposure to allow for patients starting and stopping and also changing between treatments during follow-up. The reference category for these analyses was no current use of antidepressant treatment. This category included both unexposed time in patients treated with antidepressants at other time points during follow-up, as well as unexposed time from the group of patients who did not receive any prescriptions for antidepressants during follow-up. In an additional analysis we used treatment with selective serotonin reuptake inhibitors as the reference category. We used Wald’s significance tests to identify significant differences between the antidepressant classes.

Analyses were also performed for antidepressant dose with separate dose categories within each class (≤ 0.5, > 0.5 and ≤ 1.0, and > 1.0 defined daily doses). We carried out tests for trend for each class using dose as a continuous variable. Analyses were performed for time-varying exposures of time since starting treatment (categorised as no use, 1–28 days, 29–84 days, 85 or more days) and since stopping (1–28 days, 29–84 days and 85–182 days after stopping treatment) within each antidepressant class. The 11 most commonly prescribed antidepressants were also analysed separately, first using periods of time with no antidepressant treatment as the reference category then using citalopram (the most commonly prescribed antidepressant) as the reference category, and we used Wald’s significance tests to identify significant differences between these 11 drugs. We tested for interactions between antidepressant class and age and also for the upper gastrointestinal bleed outcome we tested for interactions between antidepressant class and non-steroidal anti-inflammatory drugs and aspirin. We assessed the proportional hazards assumption using log minus log plots.

Three sensitivity analyses were performed [22]. In the first, we restricted the analyses to the first year of follow-up, since baseline characteristics are less likely to change within this period, and fewer switches occur between different antidepressant drugs, so the results are less susceptible to residual confounding. In the second sensitivity analysis, the entire follow-up period was included to increase power and encompass long durations of antidepressant use. The third sensitivity analysis used 5 years of follow-up and excluded patients who had not received any antidepressant prescriptions during follow-up. We carried out this third analysis because patients who were untreated during follow-up might differ systematically from treated patients (such as having a dislike of taking tablets, a preference for non-drug treatments or less severe depression), and these differences could distort comparisons with the untreated reference category.

We calculated absolute risks of the outcomes over 1 year based on the method described by Altman et al. [32], accounting for the confounding variables by using the adjusted hazard ratios (aHRs) from the analyses based on 1 year of follow-up.

We used all eligible patients in the database to maximise power. We used a *P* value of less than 0.01 (two-tailed) to determine statistical significance. Analyses were carried out using Stata (v12.1).

## Results

**Fig. 1:**
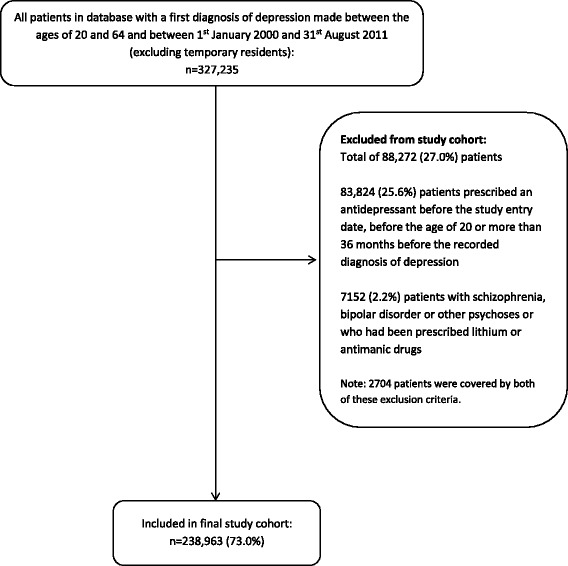
Flow chart for selection of patients included in study cohort

The initial cohort included 327,235 patients with a first diagnosis of depression made during the study period and between the ages of 20 and 64 years. A total of 88,272 (27.0%) patients were excluded because they had been prescribed an antidepressant either before the study entry date, before age 20 or more than 36 months before their date of diagnosis of depression, or had schizophrenia, bipolar disorder or other psychoses, or had been prescribed lithium or antimanic drugs. This left 238,963 eligible patients in the final study cohort (Fig. 1).

The total length of follow-up was 1,307,326 person-years, with a median of 5.2 years per person. Characteristics of the study cohort at baseline are shown in Table 1. The cohort included 146,028 (61%) women and the mean age was 39.5 (SD 11.1) years.

**Table 1:** Characteristics of the study cohort (*n* = 238,963) at baseline

Characteristic	n	%^a^
Sex		
Male	92,935	(38.9)
Female	146,028	(61.1)
Mean age (SD)	39.5	(11.1)
Ethnic group recorded	136,624	(57.2)
Ethnic group:		
White/not recorded	227,451	(95.2)
Non-white	11,512	(4.8)
Depression severity (index diagnosis):		
Mild	171,208	(71.7)
Moderate	59,140	(24.8)
Severe	8615	(3.6)
Smoking status^b^:		
Recorded	233,290	(97.6)
Non smoker	110,849	(47.5)
Ex-smoker	35,132	(15.1)
Current light smoker	24,104	(10.3)
Current moderate smoker	40,546	(17.4)
Current heavy smoker	22,659	(9.7)
Alcohol consumption^b^:		
Recorded	203,189	(85.0)
Non drinker	55,253	(27.2)
Trivial (less than 1 unit per day)	77,579	(38.2)
Light (1–2 units per day)	51,310	(25.3)
Moderate (3–6 units per day)	14,482	(7.1)
Heavy (7–9 units per day)	2174	(1.1)
Very heavy (over 9 units per day)	2391	(1.2)
Townsend deprivation score in fifths^b^:		
Recorded	230,762	(96.6)
1 (Least deprived)	45,021	(19.5)
2	46,207	(20.0)
3	48,293	(20.9)
4	47,063	(20.4)
5 (Most deprived)	44,178	(19.1)
Comorbidities at baseline:		
Any cancer	3810	(1.6)
Asthma/chronic obstructive airways disease	31,816	(13.3)
Coronary heart disease	4109	(1.7)
Diabetes	7371	(3.1)
Hypertension	17,217	(7.2)
Stroke/transient ischaemic attack	1741	(0.7)
Epilepsy/seizures	3325	(1.4)
Previous falls	4321	(1.8)
Hypothyroidism	5267	(2.2)
Obsessive-compulsive disorder	494	(0.2)
Osteoarthritis	7228	(3.0)
Osteoporosis	867	(0.4)
Liver disease	698	(0.3)
Renal disease	549	(0.2)
Rheumatoid arthritis	1301	(0.5)
Medications at baseline:		
Anticonvulsants	2672	(1.1)
Antihypertensive drugs	25,344	(10.6)
Antipsychotics	836	(0.4)
Anticoagulants	1073	(0.5)
Aspirin	7159	(3.0)
Bisphosphonates	854	(0.4)
Hypnotics/anxiolytics	11,354	(4.8)
Non-steroidal anti-inflammatory drugs	12,725	(5.3)
Statins	10,823	(4.5)
Oral contraceptives^c^	27,396	(18.8)
Hormone replacement therapy^c^	7207	(4.9)

^a^Values are numbers (column percentages) unless stated otherwise

^b^Percentages for smoking status, alcohol and deprivation are out of total with recorded values

^c^Percentage is for females only

### Antidepressant treatment during follow-up

The majority of patients in the cohort (209,476, 87.7%) were treated with antidepressants during follow-up. The median duration of treatment was 221 days (interquartile range 79–590 days), with 36.6% of treated patients having 1 or more years of treatment and 5.5% having 5 or more years of treatment. Selective serotonin reuptake inhibitors were the most frequently prescribed antidepressant class (189,968 patients had 2,379,668 prescriptions), followed by tricyclic and related antidepressants (61,901 patients had 533,798 prescriptions), other antidepressants (33,631 patients had 422,079 prescriptions), and monoamine oxidase inhibitors (156 patients had a total of 1791 prescriptions). There were 83,784 combined prescriptions where two or more different antidepressant drugs were prescribed on the same day.

The three most commonly prescribed antidepressants were citalopram (1,023,255 prescriptions; 31.5%), fluoxetine (778,285; 23.9%) and amitriptyline (236,416; 7.3%), out of a total of 3,252,633 prescriptions (with combined prescriptions counting as single prescriptions). Numbers of prescriptions overall and by prescribed daily dose category for the 11 most commonly prescribed antidepressants are shown in Additional file 1: Table S1. Prescribed doses tended to be lowest for tricyclic and related antidepressants, with the exception of lofepramine, which had the highest prescribed doses.

### Incidence rates

At baseline, 4321 patients had a previous fall recorded, 23,746 had a prior fracture recorded, 1600 had a prior upper gastrointestinal bleed, 9372 a previous road traffic accident, and 1114 a previous adverse drug reaction. These patients were excluded from analyses of each respective outcome, along with the 156 patients prescribed monoamine oxidase inhibitors.

During the first 5 years of follow-up, 4651 patients experienced one or more falls (incidence rate of 529 per 100,000 person-years), 4796 had fractures (596 per 100,000), 1066 had an upper gastrointestinal bleed (119 per 100,000), 3690 had a road traffic accident (428 per 100,000), and 1058 experienced an adverse drug reaction (118 per 100,000); further, there were 3181 deaths from all causes (351 per 100,000). In addition, 74 patients had antidepressant poisoning recorded (8 per 100,000), and there were 16 sudden deaths (2 per 100,000).

### Results of analyses for falls

Table 2 shows hazard ratios for each antidepressant class compared with periods of time when these drugs were not being used over the 5 years of follow-up. There were significantly increased rates of falls in all antidepressant classes compared with untreated periods of time. Table 3 presents HRs with selective serotonin reuptake inhibitor treatment as the reference category and shows that, in a direct comparison of fall rates between the antidepressant classes, there were no significant differences overall (*P* = 0.59). There were significant trends in fall rates by dose in each of the drug classes (Table 4).

**Table 2:** Unadjusted and adjusted hazard ratios for six adverse outcomes (falls, fracture, upper gastrointestinal bleed, road traffic accident, adverse drug reaction, and all-cause mortality) by antidepressant class compared with periods of non-use of antidepressants over 5 years follow-up

			Unadjusted analysis		Adjusted analysis^b^		
Antidepressant class	No of events^a^	Person-years^a^	Hazard ratio	95% CI	Hazard ratio	95% CI	*P*
Falls							
No current use	2493	558,933	1.00		1.00		
TCAs	298	40,260	1.79	(1.58–2.04)	1.36	(1.19–1.54)	< 0.001
SSRIs	1494	221,813	1.62	(1.52–1.73)	1.48	(1.39–1.59)	< 0.001
Other antidepressants	189	27,678	1.64	(1.42–1.89)	1.43	(1.23–1.66)	< 0.001
Combined antidepressants	37	4121	2.03	(1.48–2.78)	1.61	(1.16–2.22)	0.004
Fracture							
No current use	2759	510,172	1.00		1.00		
TCAs	205	37,309	1.04	(0.90–1.20)	0.92	(0.80–1.06)	0.26
SSRIs	1466	204,592	1.35	(1.26–1.44)	1.30	(1.21–1.39)	< 0.001
Other antidepressants	195	25,164	1.43	(1.24–1.65)	1.28	(1.11–1.48)	0.001
Combined antidepressants	29	3759	1.39	(0.94–2.06)	1.22	(0.82–1.81)	0.32
Upper GI bleed							
No current use	588	570,654	1.00		1.00		
TCAs	79	41,295	1.83	(1.45–2.29)	1.43	(1.13–1.81)	0.003
SSRIs	300	226,336	1.21	(1.05–1.39)	1.16	(1.00–1.33)	0.045
Other antidepressants	50	28,102	1.74	(1.31–2.31)	1.35	(1.00–1.82)	0.050
Combined antidepressants	12	4245	2.65	(1.49–4.69)	2.13	(1.19–3.79)	0.010
Road traffic accident							
No current use	2343	546,915	1.00		1.00		
TCAs	154	39,929	0.87	(0.72–1.05)	0.97	(0.80–1.17)	0.72
SSRIs	929	218,137	0.97	(0.89–1.05)	1.03	(0.95–1.12)	0.47
Other antidepressants	120	27,221	1.01	(0.84–1.22)	1.08	(0.90–1.30)	0.41
Combined antidepressants	18	4063	1.00	(0.64–1.56)	1.12	(0.71–1.76)	0.62
Adverse drug reaction							
No current use	432	571,401	1.00		1.00		
TCAs	122	41,380	3.11	(2.50–3.87)	2.69	(2.15–3.37)	< 0.001
SSRIs	396	226,577	1.88	(1.62–2.19)	1.75	(1.50–2.04)	< 0.001
Other antidepressants	70	28,197	3.03	(2.29–4.01)	2.81	(2.11–3.75)	< 0.001
Combined antidepressants	11	4257	3.29	(1.81–5.97)	2.92	(1.60–5.35)	0.001
All-cause mortality							
No current use	1541	575,623	1.00		1.00		
TCAs	326	41,807	2.89	(2.54–3.29)	1.92	(1.68–2.19)	< 0.001
SSRIs	990	228,233	1.63	(1.50–1.78)	1.38	(1.26–1.51)	< 0.001
Other antidepressants	196	28,487	2.61	(2.24–3.03)	1.74	(1.48–2.06)	< 0.001
Combined antidepressants	41	4299	3.62	(2.69–4.88)	2.19	(1.60–3.00)	< 0.001

^a^Based on numbers in adjusted analysis (patients with missing values for confounding variables were excluded from the adjusted analyses – during 5 years follow-up, 141 of these patients had falls, 142 had fractures, 37 had upper gastrointestinal bleeds, 126 had road traffic accidents, 27 had adverse drug reactions and 87 died)

^b^Adjusted for age, sex, year of diagnosis of depression, severity of depression, deprivation, smoking status, alcohol intake, ethnic group (white/not recorded or non-white), coronary heart disease, diabetes, hypertension, cancer, epilepsy/seizures, hypothyroidism, osteoarthritis, asthma/chronic obstructive airways disease, stroke/TIA, rheumatoid arthritis, osteoporosis, liver disease, renal disease, obsessive-compulsive disorder, statins, NSAIDS, aspirin, antihypertensive drugs, anticonvulsants, hypnotics/anxiolytics, oral contraceptives, hormone replacement therapy, antipsychotics, bisphosphonates, anticoagulants; fracture outcome also adjusted for falls

*GI gastrointestinal, SSRIs* selective serotonin reuptake inhibitors, *TCAs* tricyclic and related antidepressants

**Table 3:** Unadjusted and adjusted hazard ratios for six adverse outcomes (falls, fracture, upper gastrointestinal bleed, road traffic accident, adverse drug reaction, and all-cause mortality) by antidepressant class compared with selective serotonin reuptake inhibitors over 5 years follow-up

			Unadjusted analysis		Adjusted analysis^b^		
Antidepressant class	No of events^a^	Person-years^a^	Hazard ratio	95% CI	Hazard ratio	95% CI	*P*
Falls							
SSRIs	1494	221,813	1.00		1.00		
TCAs	298	40,260	1.11	(0.97–1.26)	0.91	(0.80–1.05)	0.20
Other antidepressants	189	27,678	1.01	(0.88–1.17)	0.96	(0.83–1.12)	0.62
Combined antidepressants	37	4121	1.25	(0.91–1.72)	1.08	(0.78–1.50)	0.63
No current use	2493	558,933	0.62	(0.58–0.66)	0.67	(0.63–0.72)	< 0.001
Comparison between groups^c^							0.59
Fracture							
SSRIs	1466	204,592	1.00		1.00		
TCAs	205	37,309	0.77	(0.67–0.89)	0.71	(0.61–0.82)	< 0.001
Other antidepressants	195	25,164	1.06	(0.91–1.24)	0.99	(0.85–1.15)	0.85
Combined antidepressants	29	3759	1.03	(0.69–1.54)	0.94	(0.63–1.40)	0.77
No current use	2759	510,172	0.74	(0.69–0.79)	0.77	(0.72–0.83)	< 0.001
Comparison between groups^c^							< 0.001
Upper GI bleed							
SSRIs	300	226,336	1.00		1.00		
TCAs	79	41,295	1.51	(1.20–1.91)	1.24	(0.97–1.57)	0.08
Other antidepressants	50	28,102	1.44	(1.08–1.93)	1.17	(0.86–1.57)	0.32
Combined antidepressants	12	4245	2.19	(1.23–3.92)	1.84	(1.03–3.30)	0.041
No current use	588	570,654	0.83	(0.72–0.95)	0.86	(0.75–1.00)	0.045
Comparison between groups^c^							0.07
Road traffic accident							
SSRIs	929	218,137	1.00		1.00		
TCAs	154	39,929	0.90	(0.74–1.09)	0.94	(0.77–1.14)	0.51
Other antidepressants	120	27,221	1.05	(0.86–1.27)	1.05	(0.87–1.27)	0.63
Combined antidepressants	18	4063	1.03	(0.66–1.62)	1.09	(0.69–1.72)	0.72
No current use	2343	546,915	1.03	(0.95–1.12)	0.97	(0.89–1.05)	0.47
Comparison between groups^c^							0.84
Adverse drug reaction							
SSRIs	396	226,577	1.00		1.00		
TCAs	122	41,380	1.65	(1.35–2.01)	1.54	(1.25–1.88)	< 0.001
Other antidepressants	70	28,197	1.61	(1.23–2.11)	1.61	(1.22–2.12)	0.001
Combined antidepressants	11	4257	1.75	(0.96–3.16)	1.67	(0.92–3.04)	0.09
No current use	432	571,401	0.53	(0.46–0.62)	0.57	(0.49–0.67)	< 0.001
Comparison between groups^c^							< 0.001
All-cause mortality							
SSRIs	990	228,233	1.00		1.00		
TCAs	326	41,807	1.77	(1.55–2.01)	1.39	(1.22–1.59)	< 0.001
Other antidepressants	196	28,487	1.59	(1.38–1.84)	1.26	(1.08–1.47)	0.003
Combined antidepressants	41	4299	2.22	(1.64–2.99)	1.58	(1.16–2.17)	0.004
No current use	1541	575,623	0.61	(0.56–0.67)	0.72	(0.66–0.79)	< 0.001
Comparison between groups^c^							< 0.001

^a^Based on numbers in adjusted analysis

^b^Adjusted for age, sex, year of diagnosis of depression, severity of depression, deprivation, smoking status, alcohol intake, ethnic group (white/not recorded or non-white), coronary heart disease, diabetes, hypertension, cancer, epilepsy/seizures, hypothyroidism, osteoarthritis, asthma/chronic obstructive airways disease, stroke/TIA, rheumatoid arthritis, osteoporosis, liver disease, renal disease, obsessive-compulsive disorder, statins, NSAIDS, aspirin, antihypertensive drugs, anticonvulsants, hypnotics/anxiolytics, oral contraceptives, hormone replacement therapy, antipsychotics, bisphosphonates, anticoagulants; fracture outcome also adjusted for falls

^c^Comparison between the different drug classes including combined antidepressants but not including no current use

*GI gastrointestinal, SSRIs* selective serotonin reuptake inhibitors, *TCAs* tricyclic and related antidepressants

**Table 4:** Unadjusted and adjusted hazard ratios for 6 adverse outcomes (falls, fracture, upper gastrointestinal bleed, road traffic accident, adverse drug reaction, and all-cause mortality) by antidepressant dose compared with periods of non-use of antidepressants over 5 years follow-up

			Unadjusted analysis		Adjusted analysis^c^		
Antidepressant class and dose categories^a^	No of events^b^	Person-years^b^	Hazard ratio	95% CI	Hazard ratio	95% CI	*P*
Falls							
No current use	2493	558,933	1.00		1.00		
*TCAs:*							
≤ 0.5 DDD	164	22,997	1.76	(1.49–2.08)	1.27	(1.07–1.50)	0.007
> 0.5 DDD/≤ 1.0 DDD	64	8191	1.88	(1.50–2.37)	1.43	(1.13–1.80)	0.003
> 1.0 DDD	46	5165	2.03	(1.50–2.75)	1.75	(1.30–2.36)	< 0.001
Test for trend^d^							0.009
*SSRIs:*							
≤ 0.5 DDD	91	15,835	1.35	(1.09–1.67)	1.19	(0.96–1.47)	0.12
> 0.5 DDD/≤ 1.0 DDD	1018	155,545	1.61	(1.49–1.73)	1.45	(1.35–1.57)	< 0.001
> 1.0 DDD	343	41,804	1.85	(1.65–2.08)	1.76	(1.56–1.98)	< 0.001
Test for trend^d^							0.001
*Others:*							
≤ 0.5 DDD	20	3967	1.14	(0.74–1.75)	1.00	(0.65–1.53)	1.00
> 0.5 DDD/≤ 1.0 DDD	86	13,046	1.62	(1.32–2.00)	1.38	(1.12–1.72)	0.003
> 1.0 DDD	68	8274	1.90	(1.51–2.39)	1.75	(1.38–2.21)	< 0.001
Test for trend^d^							0.003
Fracture							
No current use	2759	510,172	1.00		1.00		
*TCAs:*							
≤ 0.5 DDD	112	21,414	0.97	(0.81–1.16)	0.87	(0.72–1.05)	0.16
> 0.5 DDD/≤ 1.0 DDD	47	7535	1.18	(0.89–1.56)	1.01	(0.76–1.36)	0.92
> 1.0 DDD	27	4739	1.09	(0.76–1.58)	0.94	(0.65–1.38)	0.77
Test for trend^d^							0.55
*SSRIs:*							
≤ 0.5 DDD	97	14,710	1.26	(1.03–1.54)	1.21	(0.98–1.49)	0.07
> 0.5 DDD/≤ 1.0 DDD	988	143,425	1.29	(1.19–1.40)	1.24	(1.15–1.35)	< 0.001
> 1.0 DDD	336	38,450	1.61	(1.44–1.80)	1.52	(1.35–1.70)	< 0.001
Test for trend^d^							0.004
*Others:*							
≤ 0.5 DDD	25	3655	1.28	(0.86–1.91)	1.12	(0.74–1.69)	0.58
> 0.5 DDD/≤ 1.0 DDD	90	11,805	1.39	(1.12–1.72)	1.25	(1.01–1.56)	0.04
> 1.0 DDD	68	7525	1.66	(1.31–2.11)	1.48	(1.16–1.89)	0.001
Test for trend^d^							0.08
Upper GI bleed							
No current use	588	570,654	1.00		1.00		
*TCAs:*							
≤ 0.5 DDD	48	23,604	1.96	(1.45–2.64)	1.57	(1.15–2.14)	0.004
> 0.5 DDD/≤ 1.0 DDD	15	8387	1.61	(0.94–2.74)	1.26	(0.74–2.14)	0.40
> 1.0 DDD	8	5307	1.50	(0.74–3.03)	1.06	(0.50–2.27)	0.88
Test for trend^d^							0.23
*SSRIs:*							
≤ 0.5 DDD	19	16,165	1.11	(0.72–1.71)	1.20	(0.77–1.86)	0.43
> 0.5 DDD/≤ 1.0 DDD	218	158,584	1.22	(1.04–1.44)	1.20	(1.01–1.42)	0.03
> 1.0 DDD	55	42,780	1.28	(0.99–1.67)	1.08	(0.82–1.42)	0.58
Test for trend^d^							0.83
*Others:*							
≤ 0.5 DDD	5	4046	1.12	(0.46–2.70)	1.00	(0.41–2.41)	1.00
> 0.5 DDD/≤ 1.0 DDD	20	13,234	1.56	(0.98–2.47)	1.12	(0.67–1.85)	0.66
> 1.0 DDD	17	8398	2.00	(1.28–3.15)	1.54	(0.97–2.46)	0.07
Test for trend^d^							0.89
Road traffic accident							
No current use	2343	546,915	1.00		1.00		
*TCAs:*							
≤ 0.5 DDD	91	22,781	0.91	(0.74–1.13)	1.02	(0.82–1.27)	0.85
> 0.5 DDD/≤ 1.0 DDD	28	8161	0.76	(0.50–1.17)	0.84	(0.55–1.30)	0.44
> 1.0 DDD	25	5145	1.06	(0.69–1.62)	1.15	(0.75–1.76)	0.52
Test for trend^d^							0.67
*SSRIs:*							
≤ 0.5 DDD	57	15,607	0.87	(0.67–1.13)	0.94	(0.72–1.22)	0.62
> 0.5 DDD/≤ 1.0 DDD	659	152,874	0.97	(0.89–1.06)	1.04	(0.95–1.14)	0.39
> 1.0 DDD	176	41,186	0.99	(0.85–1.15)	1.03	(0.87–1.20)	0.76
Test for trend^d^							0.64
*Others:*							
≤ 0.5 DDD	14	3906	0.91	(0.53–1.55)	0.91	(0.53–1.58)	0.74
> 0.5 DDD/≤ 1.0 DDD	52	12,828	0.90	(0.68–1.19)	0.98	(0.74–1.30)	0.90
> 1.0 DDD	41	8158	1.15	(0.84–1.56)	1.23	(0.90–1.68)	0.20
Test for trend^d^							0.21
Adverse drug reaction							
No current use	432	571,401	1.00		1.00		
*TCAs:*							
≤ 0.5 DDD	71	23,632	3.10	(2.40–4.02)	2.59	(1.98–3.38)	< 0.001
> 0.5 DDD/≤ 1.0 DDD	18	8425	2.43	(1.47–4.02)	2.12	(1.28–3.51)	0.004
> 1.0 DDD	19	5320	4.03	(2.51–6.49)	3.74	(2.32–6.01)	< 0.001
Test for trend^d^							0.79
*SSRIs:*							
≤ 0.5 DDD	25	16,162	1.65	(1.09–2.49)	1.50	(0.99–2.27)	0.059
> 0.5 DDD/≤ 1.0 DDD	304	158,743	1.97	(1.66–2.33)	1.81	(1.53–2.15)	< 0.001
> 1.0 DDD	51	42,866	1.58	(1.19–2.11)	1.46	(1.08–1.97)	0.013
Test for trend^d^							0.36
*Others:*							
≤ 0.5 DDD	11	4051	3.36	(1.87–6.03)	2.79	(1.51–5.16)	0.001
> 0.5 DDD/≤ 1.0 DDD	28	13,281	2.54	(1.73–3.74)	2.35	(1.58–3.48)	< 0.001
> 1.0 DDD	18	8437	2.79	(1.72–4.53)	2.72	(1.68–4.43)	< 0.001
Test for trend^d^							0.58
All-cause mortality							
No current use	1541	575,623	1.00		1.00		
*TCAs:*							
≤ 0.5 DDD	180	23,895	2.77	(2.34–3.27)	1.77	(1.49–2.11)	< 0.001
> 0.5 DDD/≤ 1.0 DDD	69	8502	2.96	(2.30–3.80)	2.02	(1.55–2.62)	< 0.001
> 1.0 DDD	41	5363	2.89	(2.12–3.95)	1.92	(1.37–2.68)	< 0.001
Test for trend^d^							0.48
*SSRIs:*							
≤ 0.5 DDD	81	16,289	1.85	(1.49–2.31)	1.57	(1.25–1.95)	< 0.001
> 0.5 DDD/≤ 1.0 DDD	663	159,847	1.56	(1.42–1.73)	1.32	(1.19–1.47)	< 0.001
> 1.0 DDD	195	43,226	1.67	(1.43–1.94)	1.40	(1.19–1.65)	< 0.001
Test for trend^d^							0.77
*Others:*							
≤ 0.5 DDD	46	4100	4.15	(3.13–5.52)	2.59	(1.89–3.54)	< 0.001
> 0.5 DDD/≤ 1.0 DDD	87	13,404	2.48	(1.99–3.08)	1.61	(1.27–2.03)	< 0.001
> 1.0 DDD	47	8529	2.12	(1.60–2.80)	1.48	(1.10–1.99)	0.009
Test for trend^d^							0.19

^a^Daily doses could not be evaluated for some prescriptions

^b^Based on numbers in adjusted analysis

^c^Adjusted for age, sex, year of diagnosis of depression, severity of depression, deprivation, smoking status, alcohol intake, ethnic group (white/not recorded or non-white), coronary heart disease, diabetes, hypertension, cancer, epilepsy/seizures, hypothyroidism, osteoarthritis, asthma/chronic obstructive airways disease, rheumatoid arthritis, osteoporosis, liver disease, renal disease, obsessive-compulsive disorder, statins, NSAIDS, aspirin, antihypertensive drugs, anticonvulsants, hypnotics/anxiolytics, oral contraceptives, hormone replacement therapy, antipsychotics, bisphosphonates, anticoagulants; fracture outcome also adjusted for falls

^d^Test for trend uses continuous values of dose

*DDD* defined daily dose, *GI gastrointestinal, SSRIs* selective serotonin reuptake inhibitors, *TCAs* tricyclic and related antidepressants

**Fig. 2:**
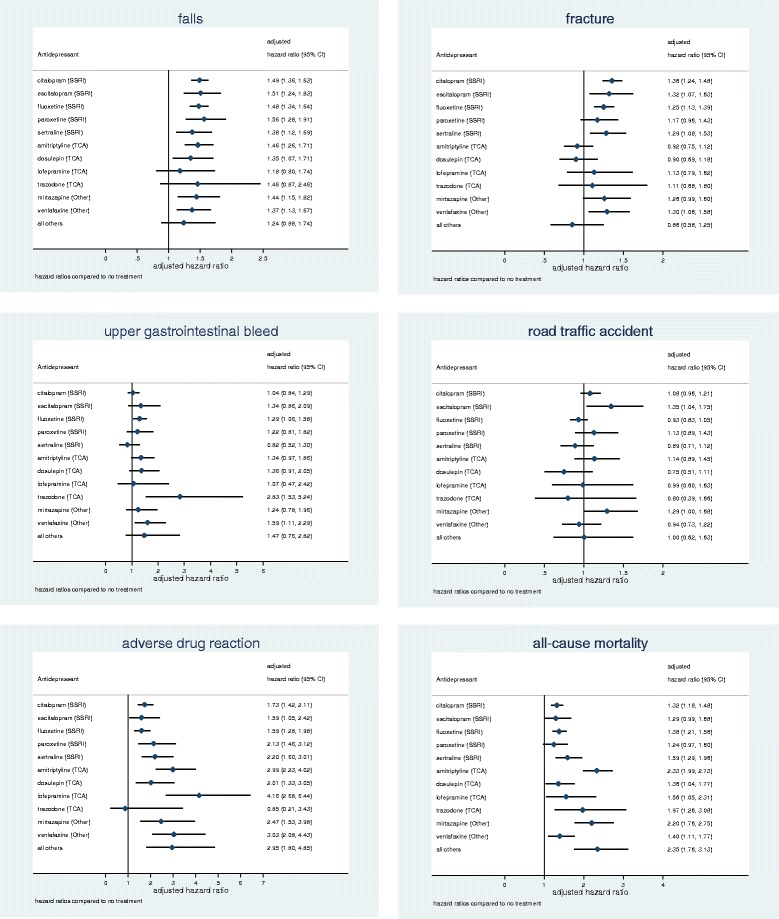
Adjusted hazard ratios for falls, fracture, upper gastrointestinal bleed, road traffic accident, adverse drug reaction, and all-cause mortality for individual antidepressant drugs over 5 years follow-up. *TCA* tricyclic and related antidepressant, *SSRI* selective serotonin reuptake inhibitor

Eight of the 11 most commonly prescribed antidepressants were associated with significantly increased fall rates (at *P* < 0.01) when compared with non-use over 5 years of follow-up (Fig. 2); for dosulepin, the association was significant at *P* = 0.013. Table 5 presents HRs with citalopram as the reference category and shows that there were no overall significant differences between the rates for these 11 drugs.

**Table 5:** Unadjusted and adjusted hazard ratios for six adverse outcomes (falls, fracture, upper gastrointestinal bleed, road traffic accident, adverse drug reaction, and all-cause mortality) by individual antidepressant drug compared with citalopram, over 5 years follow-up

			Unadjusted analysis		Adjusted analysis^b^		
Antidepressant drug	No of events^a^	Person years^a^	Hazard ratio	95% CI	Hazard ratio	95% CI	*P*
Falls							
*SSRIs:*							
Citalopram	628	92,765	1.00		1.00		
Escitalopram	92	13,180	1.04	(0.84–1.28)	1.01	(0.82–1.25)	0.91
Fluoxetine	539	80,665	1.02	(0.91–1.15)	0.99	(0.88–1.12)	0.92
Paroxetine	115	16,322	1.06	(0.87–1.30)	1.05	(0.85–1.29)	0.65
Sertraline	117	18,619	0.91	(0.74–1.13)	0.92	(0.74–1.15)	0.47
* TCAs:*							
Amitriptyline	161	18,929	1.27	(1.07–1.50)	0.98	(0.83–1.16)	0.84
Dosulepin	84	11,940	1.07	(0.83–1.37)	0.91	(0.70–1.17)	0.45
Lofepramine	27	4709	0.87	(0.59–1.29)	0.79	(0.53–1.18)	0.25
Trazodone	18	2335	1.22	(0.74–2.00)	0.98	(0.57–1.67)	0.94
*Others:*							
Mirtazapine	72	9959	1.12	(0.90–1.40)	0.97	(0.77–1.22)	0.78
Venlafaxine	97	15,489	0.91	(0.74–1.12)	0.92	(0.75–1.13)	0.44
All other antidepressants	31	4839	0.94	(0.67–1.32)	0.83	(0.59–1.18)	0.30
Combined antidepressants	37	4121	1.26	(0.91–1.74)	1.08	(0.78–1.50)	0.65
No current use	2493	558,933	0.62	(0.57–0.68)	0.67	(0.61–0.73)	< 0.001
Comparison between drugs^c^							0.98
Fracture							
*SSRIs:*							
Citalopram	645	85,297	1.00		1.00		
Escitalopram	90	12,193	0.95	(0.77–1.18)	0.97	(0.78–1.21)	0.82
Fluoxetine	509	74,542	0.90	(0.80–1.01)	0.92	(0.82–1.04)	0.19
Paroxetine	99	15,167	0.85	(0.69–1.05)	0.86	(0.70–1.07)	0.19
Sertraline	121	17,149	0.94	(0.78–1.14)	0.95	(0.78–1.15)	0.59
*TCAs:*							
Amitriptyline	98	17,646	0.73	(0.60–0.90)	0.68	(0.55–0.83)	< 0.001
Dosulepin	59	11,075	0.70	(0.53–0.91)	0.67	(0.51–0.87)	0.003
Lofepramine	28	4309	0.88	(0.62–1.25)	0.84	(0.58–1.20)	0.33
Trazodone	15	2102	0.96	(0.59–1.57)	0.82	(0.50–1.35)	0.43
*Others:*							
Mirtazapine	73	8938	1.04	(0.81–1.32)	0.93	(0.73–1.19)	0.56
Venlafaxine	106	14,174	0.96	(0.78–1.19)	0.96	(0.77–1.19)	0.68
All other antidepressants	23	4472	0.71	(0.49–1.03)	0.63	(0.43–0.93)	0.020
Combined antidepressants	29	3759	0.97	(0.65–1.45)	0.90	(0.60–1.35)	0.61
No current use	2759	510,172	0.70	(0.64–0.76)	0.74	(0.67–0.81)	< 0.001
Comparison between drugs^c^							0.015
Upper GI bleed							
*SSRIs:*							
Citalopram	110	94,862	1.00		1.00		
Escitalopram	20	13,436	1.24	(0.76–2.03)	1.29	(0.78–2.12)	0.32
Fluoxetine	123	82,236	1.28	(0.99–1.66)	1.24	(0.95–1.62)	0.11
Paroxetine	26	16,569	1.28	(0.83–1.98)	1.17	(0.75–1.82)	0.49
Sertraline	18	18,964	0.84	(0.52–1.35)	0.79	(0.48–1.30)	0.35
*TCAs:*							
Amitriptyline	35	19,565	1.60	(1.11–2.30)	1.29	(0.88–1.89)	0.19
Dosulepin	22	12,178	1.63	(1.07–2.48)	1.31	(0.85–2.02)	0.22
Lofepramine	7	4817	1.18	(0.51–2.71)	1.03	(0.45–2.36)	0.95
Trazodone	10	2343	3.94	(2.13–7.26)	2.73	(1.44–5.17)	0.002
*Others:*							
Mirtazapine	19	10,154	1.75	(1.10–2.79)	1.19	(0.73–1.95)	0.48
Venlafaxine	30	15,664	1.76	(1.19–2.58)	1.53	(1.03–2.27)	0.034
All other antidepressants	9	4945	1.71	(0.90–3.25)	1.41	(0.72–2.78)	0.31
Combined antidepressants	12	4245	2.49	(1.37–4.53)	2.04	(1.12–3.74)	0.020
No current use	588	570,654	0.94	(0.76–1.16)	0.96	(0.78–1.19)	0.73
Comparison between drugs^c^							0.08
Road traffic accident							
*SSRIs:*							
Citalopram	398	91,139	1.00		1.00		
Escitalopram	71	12,981	1.25	(0.96–1.64)	1.25	(0.95–1.64)	0.11
Fluoxetine	311	79,365	0.91	(0.78–1.05)	0.86	(0.74–1.01)	0.06
Paroxetine	78	16,030	1.11	(0.86–1.43)	1.05	(0.81–1.35)	0.73
Sertraline	69	18,362	0.87	(0.69–1.10)	0.82	(0.64–1.06)	0.13
*TCAs:*							
Amitriptyline	85	18,784	1.04	(0.80–1.36)	1.05	(0.80–1.38)	0.72
Dosulepin	35	11,855	0.68	(0.46–1.00)	0.70	(0.47–1.04)	0.08
Lofepramine	20	4694	0.93	(0.56–1.56)	0.92	(0.55–1.52)	0.74
Trazodone	7	2294	0.68	(0.33–1.41)	0.74	(0.35–1.55)	0.43
*Others:*							
Mirtazapine	50	9826	1.16	(0.88–1.52)	1.20	(0.91–1.58)	0.20
Venlafaxine	60	15,179	0.94	(0.72–1.22)	0.87	(0.67–1.14)	0.31
All other antidepressants	19	4777	0.90	(0.55–1.46)	0.93	(0.57–1.52)	0.77
Combined antidepressants	18	4063	1.01	(0.64–1.59)	1.04	(0.66–1.65)	0.87
No current use	2343	546,915	1.01	(0.90–1.13)	0.93	(0.82–1.04)	0.19
Comparison between drugs^c^							0.045
Adverse drug reaction							
*SSRIs:*							
Citalopram	163	94,933	1.00		1.00		
Escitalopram	21	13,436	0.95	(0.61–1.48)	0.92	(0.58–1.45)	0.72
Fluoxetine	134	82,362	0.95	(0.75–1.21)	0.92	(0.72–1.17)	0.51
Paroxetine	36	16,608	1.20	(0.82–1.76)	1.23	(0.83–1.82)	0.29
Sertraline	40	18,967	1.24	(0.91–1.70)	1.27	(0.92–1.74)	0.14
*TCAs:*							
Amitriptyline	66	19,635	1.94	(1.45–2.60)	1.73	(1.27–2.34)	< 0.001
Dosulepin	27	12,162	1.23	(0.80–1.88)	1.16	(0.75–1.81)	0.50
Lofepramine	21	4819	2.39	(1.55–3.70)	2.40	(1.56–3.70)	< 0.001
Trazodone	< 5	2372	0.50	(0.12–2.00)	0.49	(0.12–1.99)	0.32
*Others:*							
Mirtazapine	23	10,224	1.48	(0.92–2.37)	1.42	(0.88–2.31)	0.15
Venlafaxine	40	15,689	1.72	(1.16–2.55)	1.75	(1.17–2.62)	0.006
All other antidepressants	15	4949	1.81	(1.11–2.98)	1.71	(1.03–2.83)	0.038
Combined antidepressants	11	4257	1.78	(0.98–3.24)	1.69	(0.92–3.09)	0.09
No current use	432	571,401	0.54	(0.45–0.65)	0.58	(0.47–0.70)	< 0.001
Comparison between drugs^c^							< 0.001
All-cause mortality							
*SSRIs:*							
Citalopram	405	95,640	1.00		1.00		
Escitalopram	57	13,539	0.99	(0.77–1.26)	0.98	(0.75–1.27)	0.87
Fluoxetine	347	82,935	1.00	(0.88–1.15)	1.04	(0.91–1.20)	0.56
Paroxetine	64	16,705	0.93	(0.72–1.19)	0.94	(0.73–1.21)	0.64
Sertraline	97	19,139	1.18	(0.96–1.46)	1.21	(0.97–1.50)	0.09
*TCAs:*							
Amitriptyline	194	19,845	2.28	(1.93–2.69)	1.77	(1.49–2.10)	< 0.001
Dosulepin	64	12,292	1.23	(0.95–1.60)	1.03	(0.78–1.35)	0.83
Lofepramine	29	4863	1.42	(0.98–2.05)	1.18	(0.79–1.76)	0.42
Trazodone	24	2390	2.30	(1.47–3.60)	1.49	(0.95–2.35)	0.08
*Others:*							
Mirtazapine	110	10,343	2.54	(2.07–3.11)	1.67	(1.33–2.09)	< 0.001
Venlafaxine	73	15,835	1.11	(0.88–1.42)	1.06	(0.83–1.36)	0.65
All other antidepressants	48	5001	2.25	(1.68–3.02)	1.78	(1.32–2.38)	< 0.001
Combined antidepressants	41	4299	2.29	(1.68–3.12)	1.67	(1.21–2.31)	0.002
No current use	1541	575,623	0.63	(0.57–0.71)	0.76	(0.68–0.85)	< 0.001
Comparison between drugs^c^							< 0.001

^a^Based on numbers in adjusted analysis

^b^Adjusted for age, sex, year of diagnosis of depression, severity of depression, deprivation, smoking status, alcohol intake, ethnic group (white/not recorded or non-white), coronary heart disease, diabetes, hypertension, cancer, epilepsy/seizures, hypothyroidism, osteoarthritis, asthma/chronic obstructive airways disease, stroke/TIA, rheumatoid arthritis, osteoporosis, liver disease, renal disease, obsessive-compulsive disorder, statins, NSAIDS, aspirin, antihypertensive drugs, anticonvulsants, hypnotics/anxiolytics, oral contraceptives, hormone replacement therapy, antipsychotics, bisphosphonates, anticoagulants; fracture outcome also adjusted for falls

^c^Comparison is between the 11 individual drugs

*GI gastrointestinal, SSRIs* selective serotonin reuptake inhibitors, *TCAs* tricyclic and related antidepressants

In the analysis restricted to the first year of follow-up, aHRs for the antidepressant drug classes compared with untreated periods were smaller than in the analysis with 5 years of follow-up (Table 6).

**Table 6:** Unadjusted and adjusted hazard ratios for six adverse outcomes (falls, fracture, upper gastrointestinal bleed, road traffic accident, adverse drug reaction, and all-cause mortality) by antidepressant class compared with no antidepressant treatment, over 1 year follow-up

			Unadjusted analysis		Adjusted analysis^b^		
Antidepressant class	No of events^a^	Person-years^a^	Hazard ratio	95% CI	Hazard ratio	95% CI	*P*
Falls							
No current use	421	93,760	1.00		1.00		
TCAs	87	16,178	1.33	(1.06–1.68)	1.05	(0.83–1.33)	0.69
SSRIs	547	98,916	1.29	(1.12–1.48)	1.26	(1.10–1.45)	0.001
Other antidepressants	44	8220	1.29	(0.96–1.74)	1.15	(0.84–1.57)	0.39
Combined antidepressants	6	848	1.82	(0.88–3.76)	1.40	(0.64–3.06)	0.40
Comparison between groups^c^							0.42
Fracture							
No current use	484	85,735	1.00		1.00		
TCAs	74	14,901	0.92	(0.72–1.17)	0.81	(0.63–1.05)	0.11
SSRIs	609	90,901	1.20	(1.06–1.37)	1.16	(1.02–1.33)	0.021
Other antidepressants	62	7437	1.46	(1.12–1.90)	1.29	(0.99–1.68)	0.06
Combined antidepressants	6	779	1.33	(0.60–2.95)	1.17	(0.53–2.59)	0.70
Comparison between groups^c^							0.030
Upper GI bleed							
No current use	108	95,101	1.00		1.00		
TCAs	34	16,409	1.74	(1.21–2.49)	1.53	(1.05–2.23)	0.025
SSRIs	146	100,228	1.16	(0.91–1.50)	1.26	(0.97–1.63)	0.08
Other antidepressants	28	8282	2.92	(1.93–4.43)	2.42	(1.56–3.76)	< 0.001
Combined antidepressants	< 5	861	2.85	(0.91–8.90)	2.42	(0.80–7.28)	0.12
Comparison between groups^c^							0.006
Road traffic accident							
No current use	415	91,721	1.00		1.00		
TCAs	52	15,911	0.73	(0.52–1.02)	0.82	(0.58–1.14)	0.24
SSRIs	442	96,813	1.03	(0.91–1.18)	1.07	(0.93–1.23)	0.35
Other antidepressants	39	8037	1.11	(0.79–1.56)	1.16	(0.82–1.62)	0.41
Combined antidepressants	< 5	832	0.77	(0.25–2.36)	0.84	(0.27–2.60)	0.76
Comparison between groups^c^							0.35
Adverse drug reaction							
No current use	91	95,252	1.00		1.00		
TCAs	62	16,444	2.99	(2.15–4.17)	2.73	(1.95–3.83)	< 0.001
SSRIs	228	100,326	1.92	(1.49–2.45)	1.82	(1.41–2.34)	< 0.001
Other antidepressants	29	8316	3.35	(2.13–5.28)	3.16	(1.99–5.03)	< 0.001
Combined antidepressants	< 5	861	4.67	(1.70–12.85)	4.59	(1.66–12.71)	0.003
Comparison between groups^c^							0.005
All-cause mortality							
No current use	298	95,801	1.00		1.00		
TCAs	88	16,560	1.58	(1.23–2.04)	1.12	(0.86–1.46)	0.39
SSRIs	408	100,907	1.22	(1.05–1.41)	1.09	(0.93–1.27)	0.29
Other antidepressants	56	8380	2.01	(1.53–2.65)	1.39	(1.04–1.85)	0.024
Combined antidepressants	8	868	2.77	(1.37–5.59)	1.81	(0.86–3.81)	0.12
Comparison between groups^c^							0.22

^a^Based on numbers in adjusted analysis

^b^Adjusted for age, sex, year of diagnosis of depression, severity of depression, deprivation, smoking status, alcohol intake, ethnic group (white/not recorded or non-white), coronary heart disease, diabetes, hypertension, cancer, epilepsy/seizures, hypothyroidism, osteoarthritis, asthma/chronic obstructive airways disease, stroke/TIA, rheumatoid arthritis, osteoporosis, liver disease, renal disease, obsessive-compulsive disorder, statins, NSAIDS, aspirin, antihypertensive drugs, anticonvulsants, hypnotics/anxiolytics, oral contraceptives, hormone replacement therapy, antipsychotics, bisphosphonates, anticoagulants; fracture outcome also adjusted for falls

^c^Comparison between the four different drug groups including combined antidepressants but not no current use

*GI gastrointestinal, SSRIs* selective serotonin reuptake inhibitors, *TCAs* tricyclic and related antidepressants

### Results of analyses for fracture

Over 5 years of follow-up, the fracture rate was significantly increased for selective serotonin reuptake inhibitors (aHR 1.30, 95% CI 1.21–1.39) and other antidepressants (1.28, 95% CI 1.11–1.48), but not tricyclic and related antidepressants (0.92, 95% CI 0.80–1.06) when compared with periods of time when antidepressants were not being used (Table 2). There was a significantly lower fracture rate for tricyclic and related antidepressants when directly compared with selective serotonin reuptake inhibitors (aHR 0.71, 95% CI 0.61–0.82) with significant differences (*P* < 0.001) between the antidepressant drug classes overall (Table 3). Fracture rates increased significantly with dose only for selective serotonin reuptake inhibitors (Table 4).

There were significantly increased fracture rates for citalopram, escitalopram, fluoxetine, sertraline and venlafaxine when compared with no use of antidepressants over 5 years of follow-up (Fig. 2). In a direct comparison with citalopram as the reference category, fracture rates were significantly reduced for amitriptyline (aHR 0.68, 95% CI 0.55–0.83) and dosulepin (aHR 0.67, 95% CI 0.51–0.87) (Table 5), with some indication of significant differences between the 11 most commonly prescribed antidepressants (*P* = 0.015).

aHRs for the antidepressant drug classes compared with untreated periods in two separate age groups (20–44, 45–64 years) are shown in Additional file 1: Table S2; there was a significant interaction between drug class and age (*P* = 0.01). For the group of other antidepressants there was a significantly increased risk in people aged 20–44 (aHR 1.50, 95% CI 1.25–1.80) but no significant association in people aged 45–64 (aHR 1.04, 95% CI 0.82–1.32). Selective serotonin reuptake inhibitors were associated with a significantly increased risk of fracture in both age groups. aHRs for selective serotonin reuptake inhibitors compared with untreated periods were similar in men (1.31, 95% CI 1.18–1.45) and women (1.30, 95% CI 1.19–1.42).

### Results of analyses for upper gastrointestinal bleed

Rates of upper gastrointestinal bleed over 5 years of follow-up were significantly increased for tricyclic and related antidepressants and combined antidepressants compared with no use of antidepressants (Table 2), although they were not significantly increased when directly compared with selective serotonin reuptake inhibitors (Table 3). Further, there were no significant differences between the antidepressant drug classes overall (*P* = 0.07) and no significant trends with dose (Table 4).

Trazodone was associated with a significantly increased rate of upper gastrointestinal bleeds compared with no use of antidepressants over 5 years of follow-up (Fig. 2). In a direct comparison with citalopram as the reference group, the aHR for trazodone was 2.73 (95% CI 1.44–5.17), and there was also some indication of an increased risk for venlafaxine (aHR 1.53, 95% CI 1.03–2.27), although there were no significant overall differences between the 11 most commonly prescribed antidepressants (*P* = 0.08) (Table 5).

HRs for selective serotonin reuptake inhibitors, other antidepressants and combined antidepressants compared with untreated periods were higher in people aged 20–44 than people aged 45–64 (Additional file 1: Table S2), but the interaction between drug class and age was not statistically significant (*P* = 0.11). There were no significant interactions between antidepressant drug class and use of either non-steroidal anti-inflammatory drugs or aspirin.

In the analysis restricted to the first year of follow-up, the aHR for the group of other antidepressants compared with untreated periods was higher than in the 5-year analysis (aHR 2.42, 95% CI 1.56–3.76 for 1 year analysis) but the aHRs for the other drug classes were similar in both analyses (Table 6). HRs for the individual drugs when compared with citalopram were similar in the 1- and 5-year analyses, except for venlafaxine, where the aHR was higher in the analysis restricted to the first year of follow-up than in the 5-year analysis (aHR 3.10, 95% CI 1.88–5.11) (Additional file 1: Table S3), and there were significant differences between the 11 most commonly prescribed antidepressants (*P* = 0.002).

### Results of analyses for road traffic accidents

There were no significant associations between road traffic accidents and the antidepressant drug classes (Table 2) or individual drugs (Fig. 2 and Table 5), and no significant trends with dose (Table 4).

### Results of analyses for adverse drug reactions

Rates of adverse drug reactions over 5 years of follow-up were significantly increased for all classes of antidepressants compared with non-use (Table 2). There were significant differences between the classes overall, with higher rates for tricyclic and related antidepressants and other antidepressants when directly compared with selective serotonin reuptake inhibitors (aHRs 1.54 (95% CI 1.25–1.88) and 1.61 (1.22–2.12), respectively; Table 3), but no significant trends with dose in any of the drug classes (Table 4).

Most of the 11 most commonly prescribed antidepressants were associated with significantly increased risks (at *P* < 0.01) compared with non-use over 5 years of follow-up (Fig. 2), with the exception of trazodone (*P* = 0.82) and escitalopram (*P* = 0.03). There were significant overall differences between the most commonly prescribed antidepressants with significantly higher rates for amitriptyline, lofepramine and venlafaxine when compared with citalopram as the reference category (Table 5).

There was a significant interaction between antidepressant drug class and age (*P* < 0.001) with higher aHRs in people aged 20–44 than those aged 45–64 years for all drug classes when compared with untreated periods over 5 years of follow-up (Additional file 1: Table S2).

### Results of analyses for all-cause mortality

In the analysis of 5 years of follow-up, all-cause mortality rates were significantly increased for all classes of antidepressants compared with non-use (Table 2). The reductions in HRs comparing unadjusted and adjusted results were mainly due to adjustment for age, with some additional decreases from adjusting for use of other drugs. When directly compared with selective serotonin reuptake inhibitors, mortality rates were significantly higher for tricyclic and related antidepressants, other antidepressants and combined antidepressants (aHRs 1.39 (95% CI 1.22–1.59), 1.26 (1.08–1.47) and 1.58 (1.16–2.17), respectively; Table 3) with significant differences between the drug classes. There were no significant trends with dose in any of the drug classes (Table 4).

HRs for the 11 most commonly prescribed antidepressants compared with non-use are shown in Fig. 2. In the analysis with citalopram as the reference group, there were significantly higher mortality rates for amitriptyline (aHR 1.77, 95% CI 1.49–2.10) and mirtazapine (aHR 1.67, 95% CI 1.33–2.09; Table 5), with significant differences between the 11 most commonly prescribed antidepressants overall (*P* < 0.001).

In the analysis restricted to the first year of follow-up, there were no significant increases in mortality rates for any of the drug classes compared with non-use (at *P* < 0.01; Table 6), and HRs for tricyclic and related antidepressants, other antidepressants and combined antidepressants were no longer significantly increased when compared with selective serotonin reuptake inhibitors (aHRs 1.03 (95% CI 0.80–1.32), 1.28 (0.97–1.68) and 1.67 (0.80–3.49), respectively).

For individual drugs during the first year of follow-up the HR for amitriptyline was lower than in the 5-year analysis and was not associated with a significantly increased mortality rate compared with citalopram (1.36, 95% CI 0.99–1.86), but the HR for mirtazapine was almost the same as in the 5-year analysis (aHR 1.63, 95% CI 1.12–2.38, *P* = 0.011; Additional file 1: Table S3), although numbers were smaller.

### Analyses of duration of use

HRs according to time since starting and stopping treatment for each antidepressant class over 5 years of follow-up are shown in Additional file 1: Table S4. These show that, generally, rates remained increased throughout treatment for all classes of antidepressants for falls. For fractures, the rates were significantly increased after 28 days of starting selective serotonin reuptake inhibitor treatment and during the 28–84 days after starting treatment with the group of other antidepressants. For adverse drug reactions, rates were highest during the first 28 days of treatment but remained increased throughout treatment for all antidepressant classes. All-cause mortality rates were only significantly increased during the first 28 days of treatment for all antidepressant classes, and were significantly reduced after treatment of 85 or more days with selective serotonin reuptake inhibitors. All-cause mortality rates were highest during the first 1–28 days after stopping treatment.

### Sensitivity analyses

There were some differences between the results of analyses restricted to 1 year of follow-up and the main 5-year analyses as described above. When the entire follow-up period was used, and when patients who had not received any antidepressant prescriptions during follow-up were removed from the analysis, the aHRs comparing antidepressant classes with untreated periods were similar to those in the main 5-year analyses for all outcomes (Additional file 1: Table S5).

### Absolute risks

Table 7 shows absolute risks of the six outcomes over 1 year by antidepressant class and for the individual drugs. Absolute risks were mostly less than 60 per 10,000 patients over 1 year and were highest overall for falls and fractures. The absolute risk of fracture associated with selective serotonin reuptake inhibitors was 20 per 10,000 higher than for tricyclic and related antidepressants, and for other antidepressants it was 27 per 10,000 higher. The absolute risk of a gastrointestinal bleed was 29 per 10,000 higher for venlafaxine compared with citalopram. Mirtazapine was associated with an excess risk of 23 per 10,000 for all-cause mortality compared with citalopram.

**Table 7:** Absolute risks of falls, fracture, upper gastrointestinal bleed, road traffic accident, adverse drug reaction, and all-cause mortality over 1 year by antidepressant class and for individual antidepressant drugs

	Absolute risks (per 10,000 persons) over 1 year^a,b^					
	Falls	Fracture	Upper GI bleed	Road traffic accident	Adverse drug reaction	All-cause mortality
Antidepressant class						
SSRIs	57	66	15	49	18	36
TCAs	47	46	18	38	27	37
Other antidepressants	52	73	29	53	32	46
Combined antidepressants	63	67	29	39	46	60
Antidepressant drug						
*SSRIs:*						
Citalopram	56	66	13	50	16	36
Escitalopram	66	65	19	62	21	38
Fluoxetine	57	66	17	43	16	35
Paroxetine	60	71	17	61	26	29
Sertraline	52	66	5	55	28	38
*TCAs:*						
Amitriptyline	59	48	19	41	25	49
Dosulepin	45	52	15	29	22	22
Lofepramine	30	38	16	39	56	45
Trazodone	33	34	38	43	c	23
*Others:*						
Mirtazapine	62	60	19	56	27	59
Venlafaxine	43	81	42	47	38	34
All other antidepressants	29	70	23	72	32	52
No treatment	45	57	12	46	10	33

^a^Absolute risks are adjusted for confounders listed in Table 2

^b^Absolute risks over 1 year evaluated using adjusted hazard ratios from analyses restricted to 1 year of follow-up

^c^Insufficient numbers for calculation

*SSRIs* selective serotonin reuptake inhibitors, *TCAs* tricyclic and related antidepressants

## Discussion

This large study found several differences in the rates of adverse outcomes between different antidepressant classes and individual drugs in people aged 20–64 years with a diagnosis of depression. Our key findings were that selective serotonin reuptake inhibitors and other antidepressants were associated with significantly increased fracture rates. All drug classes were associated with significantly increased risks of falls. Rates of adverse drug reaction were significantly higher for tricyclic and related antidepressants and other antidepressants than for selective serotonin reuptake inhibitors. Mortality rates were significantly higher for tricyclic antidepressants and other antidepressants than with selective serotonin reuptake inhibitor treatment over 5 years of follow-up, but not during the first year of follow-up.

Among individual antidepressant drugs, fracture rates were significantly increased for citalopram, escitalopram, fluoxetine, sertraline and venlafaxine compared with periods of non-use of antidepressants. Amitriptyline, lofepramine and venlafaxine were associated with significantly higher rates of adverse drug reactions compared with citalopram. Trazodone was associated with a significantly higher rate of upper gastrointestinal bleeding over 5 years of follow-up. Mirtazapine and amitriptyline were associated with highest mortality rates over 5 years of follow-up, but only mirtazapine was associated with a significantly increased risk during the first year of follow-up.

In this cohort of adults aged 20–64 years, the absolute risks of the adverse outcomes were mostly less than 0.6% per year, and for falls, fractures, upper gastrointestinal bleeding and all-cause mortality they were considerably lower than the equivalent risks in older people [26]. Whilst for individuals the excess risks associated with antidepressant use are low, given the widespread use of these drugs in adults, the population effects could be considerable.

Additional analyses examining patterns of risk according to duration of use found the increases in all-cause mortality rates across all antidepressant classes were only apparent during the first 28 days of treatment, after which they declined rapidly. This is a period during which depressive symptoms can be most severe, and we have previously shown that rates of suicide and self-harm in this cohort were highest in the first 28 days after starting antidepressant treatment [22]. Mortality rates were also substantially increased in the first 28 days after stopping antidepressants, which may reflect patients stopping medication due to onset of illness or hospital or hospice admission rather than a direct effect of drug withdrawal. Although amitriptyline was associated with the highest increase in mortality rates over 5 years of follow-up it was not associated with a significantly increased risk during the first year of follow-up. The increased risk over 5 years of follow-up might occur due to amitriptyline being initiated to relieve neuropathic pain in patients who developed cancer although it is not specifically licenced for this in the UK [33]. However, the increased mortality rate for mirtazapine was similar in magnitude in the 1- and 5-year analyses.

### Strengths and limitations

This study included a large representative sample of 238,963 people aged 20–64 diagnosed with depression in the general UK population. All eligible patients were included, so there is no selection bias arising from non-response. Data on prescriptions and confounding variables were recorded prospectively before the outcomes occurred, thereby avoiding recall bias.

To reduce indication bias we only included patients with a diagnosis of depression, since antidepressants, and particularly tricyclic antidepressants, are prescribed for a range of indications, including off-label conditions such as insomnia and pain, and these indications will be associated with the outcomes considered in this study to a varying degree [34]. Depression itself is an established risk factor for several of the outcomes considered here, including falls, fracture and all-cause mortality [35–39], and restricting the cohort to patients with a diagnosis of depression helped distinguish the effects of antidepressant treatment from those of depression itself. We also adjusted for severity of depression at first diagnosis, although we were not able to account for changes in severity of depression over time as depression severity scores are not routinely recorded in general practice. Our cohort only included people with a first diagnosis of depression who had not previously been prescribed antidepressants to avoid biases associated with prevalent use or prior experiences during previous treatment episodes [40]. The results of the sensitivity analyses excluding patients who did not receive antidepressant prescriptions during follow-up were very similar to the main analyses where these patients contributed follow-up time to the unexposed reference category. This indicates that including these patients who may differ in terms of treatment preferences and other personal characteristics did not distort the results.

We carried out analyses directly comparing event rates during treatment with different antidepressant classes as well as including comparisons with untreated periods. Comparisons with untreated periods of time are still susceptible to indication bias since the depression may have resolved or be less severe during these periods, leading to a reduced incidence of the events. Further, this could explain the increased rates of mortality during periods of treatment with all classes of antidepressant compared with untreated periods, particularly in the 5-year analysis, where patients receiving antidepressant treatment after 1 year are likely to have more severe or treatment-resistant depression. The analyses directly comparing treated groups with each other are less vulnerable to these biases.

We accounted for a large number of potential confounding variables in the analysis, including other comorbidities and use of other medications; however, as with any observational study, the findings are still susceptible to residual confounding due to lack of adjustment for certain potential risk factors such as dietary factors and physical activity, which are not routinely recorded in primary care. Similarly, we did not adjust for chronic pain, since it is inconsistently recorded in primary care, or for conditions such as multiple sclerosis and fibromyalgia, but these would likely have a low prevalence in this age group.

Our outcomes were restricted to medical outcomes recorded in GP records or on death certificates, and we were not able to include pertinent outcomes such as interpersonal and psychological effects as they are seldom included in these records [41]. A further limitation is that the outcomes were not formally adjudicated in this study, and some more minor events, such as might occur for falls, adverse drug reactions or road traffic accidents, would not be medically reported or recorded so there will be some misclassification of the outcomes; this also means our findings for these outcomes relate to more severe, medically reported events. Validation studies in other UK primary care databases have shown high levels of validity across a range of diseases; for example, Khan [42] reported high positive predictive values for validation studies of upper gastrointestinal bleeding and hip fracture. We included information from death certificates to identify additional patients with the outcomes, which will have increased ascertainment and reduced misclassification. However, most codes used to identify road traffic accidents did not indicate whether the person was driving or a passenger, or whether they were responsible for the crash; therefore, findings for this outcome are particularly susceptible to misclassification biases. We excluded patients with a prior history of each adverse outcome from the analysis of that outcome to ensure that only new events were included and to remove potential biases arising from changes to treatments and behaviours as a consequence of prior events.

There is likely to be some misclassification of the antidepressant exposure variables as patients may not have actually taken their prescribed antidepressant medication, or may not have taken it at the prescribed dose. This misclassification could underestimate associations with drug use. Furthermore, although the cohort was large, the number of events was small for some of the antidepressant exposure categories and some of the stratified analyses.

### Comparison with other studies

Many observational studies have consistently found increased risks of falls in older people taking antidepressants [26, 43–45]. Fewer studies have examined the risks in younger people. Our findings show that rates of falls are also increased in younger people taking antidepressants, and increase with dose. A review of studies in older people found that the increased risks of falls were similar for selective serotonin reuptake inhibitors and tricyclic antidepressants [14]; likewise, we found associations were similar for these drug classes in younger people. A number of factors are likely to explain the increased risk of falls associated with antidepressants, including effects on concentration, balance and reaction times, and orthostatic hypertension and sedative effects, particularly for the tricyclic antidepressants and mirtazapine, and sleep disturbance with selective serotonin reuptake inhibitors resulting in drowsiness and dizziness [14].

Our finding of an increased risk of fracture for selective serotonin reuptake inhibitors concurs with the findings of many other observational studies [15, 46], though these have predominantly been conducted in older populations, whilst we found an increased risk even in people aged 20–44. These increases may be due to decreased bone density since use of selective serotonin reuptake inhibitors has been shown to be associated with a reduction in bone mineral density and bone loss, even in adolescent boys [47]. We did not find an increased fracture risk for tricyclic antidepressants, which contrasts with the findings of two systematic reviews [15, 48], although these found smaller increases for tricyclic antidepressants than for selective serotonin reuptake inhibitors [46]. A Danish case-control study [49] that examined age and dose effects for selective serotonin reuptake inhibitors and tricyclic antidepressants found that the fracture risk associated with selective serotonin reuptake inhibitors increased with age but only in medium- and high-dose users, whilst for tricyclic antidepressants there was only an increased fracture risk in the oldest age group (> 60 years) for the highest dose. Few studies have examined other antidepressants or individual antidepressants, yet a recent cohort study of middle-aged and older adults found similar fracture risks when comparing serotonin-norepinephrine reuptake inhibitors (venlafaxine and duloxetine) with selective serotonin reuptake inhibitors [50].

Several studies have found upper gastrointestinal bleeding to be more common among patients taking selective serotonin reuptake inhibitors, particularly when used in combination with non-steroidal anti-inflammatory drugs [17, 51]. A number of mechanisms have been proposed for this increased risk, including depletion of platelet serotonin content causing an inhibition of platelet plug formation or direct toxicity on the gastrointestinal mucosa [52]. In our study, we found a higher risk for tricyclic and related antidepressants than for selective serotonin reuptake inhibitors, although this was only in the lowest dose category and may therefore reflect preferential prescribing of low-dose tricyclic antidepressants rather than selective serotonin reuptake inhibitors in people with suspected risk factors for bleeding. We did not find a stronger association when selective serotonin reuptake inhibitors were used in combination with non-steroidal anti-inflammatory drugs, although, as our study was in a younger age group than most previous studies, these differences may be due to smaller numbers prescribed this drug combination. Our finding that venlafaxine and trazodone were associated with the highest risks has been found in other studies [26, 53–55].

Increased tolerability of selective serotonin reuptake inhibitors in comparison with tricyclic antidepressants is long established [56–58], with selective serotonin reuptake inhibitors having fewer side effects and adverse reactions, particularly for anticholinergic and sedating effects. Lofepramine had the highest rate of adverse drug reactions in this study, as we also found in our study of older people [26], though this drug was prescribed at higher doses than the other antidepressants. Venlafaxine was also associated with an increased risk of adverse drug reactions compared with citalopram, which concurs with the findings of a meta-analysis of double-blind randomized trials that reported higher rates of discontinuation due to adverse events for venlafaxine compared with selective serotonin reuptake inhibitors [59]. Amitriptyline was mainly prescribed at low doses, but still showed an increased risk of adverse drug reactions.

We found no evidence of associations with antidepressant treatment for road traffic accidents, although our outcome was broad and not specific to drivers of vehicles. Findings from other studies are inconclusive, with some showing increased risks for selective serotonin reuptake inhibitors and some for tricyclic antidepressants particularly in older people, whilst others have shown no associations with antidepressant use [16, 60–64]. Many of these studies have not accounted for depression, which itself can impair driving performance [65, 66].

Our findings of increased mortality rates over 5 years for all antidepressant classes are similar to those of a cohort study in postmenopausal women that found increased mortality rates among users of selective serotonin reuptake inhibitors, tricyclic and related antidepressants, and other antidepressants with a mean follow-up of 5.9 years [19]. The authors proposed possible mechanisms for these associations but also suggested they could be due to antidepressant use reflecting other causes of increased mortality, such as residual depressive symptoms, that may not have been fully controlled. Previous observational studies in people aged 65 and over have found mirtazapine to be associated with the highest increases in mortality rates [26, 67]. A study of FDA Summary Basis of Approval reports was carried out to assess whether medication may worsen the already increased mortality risk for patients with severe psychiatric illness [38]. This study combined mortality rates across short- or medium-term randomised clinical trials of psychotropic drugs in patients with psychiatric illness, and found that, among patients with depression, the overall mortality risk was similar for selective serotonin reuptake inhibitors or selective serotonin-norepinephrine reuptake inhibitors compared to placebo, but there was a significantly higher risk for the group of heterocyclic antidepressants, which included amitryptiline, imipramine, maprotiline and mirtazapine. Suicide was the most common cause of death. This study of trial data, which is not susceptible to residual confounding, provides some support for our findings of increased mortality rates for amitryptiline and mirtazapine in comparison with citalopram, although the study did not assess these drugs individually and the number of deaths was low. Herein, we have not investigated specific causes of death, although in an analysis of suicide we found a 3.7-fold increased risk for mirtazapine but no association for amitryptiline [22], although suicide only accounted for a relatively small proportion of all deaths.

### Clinical implications and future research

Antidepressants are one of the most commonly prescribed medications in younger and middle-aged adults, although their benefits in the treatment of depression may be relatively small, especially for mild and moderate depression [10, 68]. These small beneficial effects could be outweighed by harmful effects, but there is limited evidence on their safety in younger and middle-aged adults, particularly for outcomes such as falls and fracture. Although susceptible to indication bias and residual confounding, this study has found increased rates of falls, fractures, upper gastrointestinal bleeds, adverse drug reactions and all-cause mortality during periods of antidepressant use compared with non-use for most classes of antidepressant. This study also found that, over 5 years, selective serotonin reuptake inhibitors and other antidepressants were associated with significantly increased fracture rates compared with tricyclic and related antidepressants, whereas rates of all-cause mortality and adverse drug reaction were significantly higher for tricyclic and related antidepressants and other antidepressants than for selective serotonin reuptake inhibitors. These risks should be carefully considered and balanced against potential benefits for individual patients when the decision to prescribe an antidepressant is made so as to avoid unnecessary treatment or to help select the most appropriate treatment where required.

Of particular concern is the association of mirtazapine with increased suicide and mortality rates in each of the observational studies that we have performed [22, 26] and in the US study of randomised controlled trial reports [38], whereas other antidepressant drugs have shown inconsistent relationships with mortality risk. This relationship, along with the increased risk for amitriptyline over 5 years, requires further investigation to ascertain how much of the increased risk for mortality is associated with suicide and other specific causes of death, how much due to selective prescribing in people with life threatening illness (such as because of the sedation it produces to aid sleep in people who might have pain as well as depression) and how much is due to some other mechanism.

## Conclusions

This large study of potential adverse outcomes in combination with the findings for cardiovascular outcomes [24], suicide and self-harm [22], and epilepsy [23] has provided a comprehensive assessment of antidepressant safety in people aged 20–64 years diagnosed with depression. Although the findings are from an observational study design and are therefore susceptible to residual confounding, our results do indicate potential increased risks for some adverse outcomes for consideration when antidepressants are prescribed. Thus, even though they are quite rare outcomes, these adverse effects of antidepressants need to be considered alongside the benefits in working age adults as well as in older people.

## Additional file

Additional file 1: **Table S1.** Numbers of prescriptions for different antidepressant drugs by dose category. **Table S2.** Adjusted hazard ratios for six adverse outcomes (falls, fracture, upper gastrointestinal bleed, adverse drug reaction, road traffic crash and all-cause mortality) by antidepressant class compared with no use of antidepressants in (A) ages 20–44 years and (B) 45–64 years over 5 years of follow-up. **Table S3.** Unadjusted and adjusted hazard ratios for six adverse outcomes (falls, fracture, upper gastrointestinal bleed, adverse drug reaction, road traffic crash and all-cause mortality) by antidepressant drug, compared with citalopram over 1 year of follow-up. **Table S4.** Adjusted hazard ratios for six adverse outcomes (falls, fracture, upper gastrointestinal bleed, adverse drug reaction, road traffic crash and all-cause mortality) by antidepressant class compared with no use of antidepressants according to duration of use and time since stopping for each antidepressant class over 5 years of follow-up. **Table S5.** Adjusted hazard ratios for six adverse outcomes (falls, fracture, upper gastrointestinal bleed, adverse drug reaction, road traffic crash and all-cause mortality) by antidepressant class compared with no use of antidepressants, over (A) total follow-up time and (B) 5 years of follow-up, excluding untreated patients. (DOCX 115 kb)

## Abbreviations

aHR: Adjusted hazard ratio
CI: Confidence interval
EMIS: Egton Medical Information Systems
HR: Hazard ratio
