# Supplementary material for: Antidepressant use and risk of adverse outcomes in people aged 20–64 years: cohort study using a primary care database
Source: BMC Med. 2018 Mar 8;16:36. doi: 10.1186/s12916-018-1022-x (PMC5842559; doi:10.1186/s12916-018-1022-x)
Supplement: Supplementary file 1 — Table S1. Numbers of prescriptions for different antidepressant drugs by dose category. Table S2. Adjusted hazard ratios for six adverse outcomes (falls, fracture, upper gastrointestinal bleed, adverse drug reaction, road traffic crash and all-cause mortality) by antidepressant class compared with no use of antidepressants in (A) ages 20–44 years and (B) 45–64 years over 5 years of follow-up. Table S3. Unadjusted and adjusted hazard ratios for six adverse outcomes (falls, fracture, upper gastrointestinal bleed, adverse drug reaction, road traffic crash and all-cause mortality) by antidepressant drug, compared with citalopram over 1 year of follow-up. Table S4. Adjusted hazard ratios for six adverse outcomes (falls, fracture, upper gastrointestinal bleed, adverse drug reaction, road traffic crash and all-cause mortality) by antidepressant class compared with no use of antidepressants according to duration of use and time since stopping for each antidepressant class over 5 years of follow-up. Table S5. Adjusted hazard ratios for six adverse outcomes (falls, fracture, upper gastrointestinal bleed, adverse drug reaction, road traffic crash and all-cause mortality) by antidepressant class compared with no use of antidepressants, over (A) total follow-up time and (B) 5 years of follow-up, excluding untreated patients. (DOCX 115 kb) [file 12916_2018_1022_MOESM1_ESM.docx]

**Antidepressant use and risk of adverse in people aged 20 to 64: cohort study using a primary care database**

**ADDITIONAL TABLES**

Table S1 Numbers of prescriptions for different antidepressant drugs by dose category

|  |  |  |  | **Daily prescribed dose (DDD categories)^3^** | | |
| --- | --- | --- | --- | --- | --- | --- |
| **Antidepressant drug** | **n^1^** | **%^2^** | **DDD value**  **(mg/day)** | **≤ 0.5 DDD** | **>0.5 DDD &**  **≤ 1.0 DDD** | **>1.0 DDD** |
|  |  |  |  | **n (row%)** | **n (row%)** | **n (row%)** |
|  |  |  |  |  |  |  |
| *Tricyclic and related antidepressants (TCA)* | | |  | 256,326 (59.6) | 107,027 (24.9) | 66,991 (15.6) |
| Amitriptyline | 236,416 | 7.3 | 75 | 141,234 (65.6) | 50300 (23.4) | 23,897 (11.1) |
| Dosulepin | 125,302 | 3.9 | 150 | 77,952 (66.4) | 36235 (30.9) | 3269 (2.8) |
| Lofepramine | 47,414 | 1.5 | 105 | 134 (0.3) | 7973 (18.4) | 35,242 (81.3) |
| Trazodone | 30,912 | 1.0 | 300 | 22,367 (78.4) | 5827 (20.4) | 324 (1.1) |
|  |  |  |  |  |  |  |
| *Selective serotonin reuptake inhibitors (SSRI)* | | |  | 157,054 (7.0) | 1,530,425 (68.5) | 545,365 (24.4) |
| Citalopram | 1,023,255 | 31.5 | 20 | 134,050 (13.6) | 616,489 (62.7) | 233,413 (23.7) |
| Escitalopram | 139,190 | 4.3 | 10 | 9428 (7.0) | 78,200 (58.0) | 47,150 (35.0) |
| Fluoxetine | 778,285 | 23.9 | 20 | 5810 (0.8) | 613,097 (81.2) | 135,992 (18.0) |
| Paroxetine | 159,389 | 4.9 | 20 | 5997 (3.9) | 106,405 (69.8) | 39,960 (26.2) |
| Sertraline | 213,749 | 6.6 | 50 | 1373 (0.7) | 115,060 (56.2) | 88,325 (43.1) |
|  |  |  |  |  |  |  |
| *Other antidepressants* |  |  |  | 49,518 (14.0) | 167,833 (47.3) | 137,453 (38.7) |
| Mirtazapine | 142,400 | 4.4 | 30 | 34,150 (25.4) | 64,031 (47.6) | 36,468 (27.1) |
| Venlafaxine | 205,984 | 6.3 | 100 | 11,298 (5.8) | 86,433 (44.1) | 98,457 (50.2) |
|  |  |  |  |  |  |  |
| All other antidepressants | 66,553 | 2.0 | - |  |  |  |
| Combined antidepressants^4^ | 83,784 | 2.6 | - |  |  |  |
|  |  |  |  |  |  |  |
| Total prescriptions | 3,252,633 |  |  |  |  |  |
|  |  |  |  |  |  |  |

^1^ Number of prescriptions, where prescriptions for the same drug issued on the same day count as a single prescription and the doses have been summed.

^2^ Column percentage out of total number of antidepressant prescriptions= 3,252,633

^3^ Prescribed daily doses could not be evaluated for some prescriptions.

^4^ Combined prescriptions for different antidepressant drugs are considered as a single prescription in this table.

DDD = defined daily dose value for the antidepressant drug

Table S2 Adjusted hazard ratios for six adverse outcomes (falls, fracture, upper gastrointestinal bleed, adverse drug reaction, road traffic crash, and all-cause mortality) by antidepressant class compared with no use of antidepressants in (A) ages 20 to 44 years and (B) 45 to 64 years over 5 year’s follow-up.

|  | 1. Ages 20 to 44 years | | | | | 1. Ages 45 to 64 years | | | | |
| --- | --- | --- | --- | --- | --- | --- | --- | --- | --- | --- |
| **Antidepressant class** | No of events^*^ | Person years^*^ | Adjusted hazard ratio† | 95% CI | p | No of events^*^ | Person years^*^ | Adjusted hazard ratio† | 95% CI | P |
| **FALLS** |  |  |  |  |  |  |  |  |  |  |
| No current use | 1373 | 384,886 | 1.00 |  |  | 1120 | 174,047 | 1.00 |  |  |
| TCAs | 116 | 22,035 | 1.33 | (1.08 to 1.62) | 0.006 | 182 | 18,225 | 1.40 | (1.19 to 1.63) | <0.001 |
| SSRIs | 701 | 142,227 | 1.40 | (1.28 to 1.54) | <0.001 | 793 | 79,586 | 1.59 | (1.44 to 1.75) | <0.001 |
| Other antidepressants | 80 | 16,974 | 1.28 | (1.03 to 1.60) | 0.027 | 109 | 10,704 | 1.58 | (1.29 to 1.92) | <0.001 |
| Combined antidepressants | 13 | 2,298 | 1.35 | (0.77 to 2.38) | 0.29 | 24 | 1,823 | 1.82 | (1.22 to 2.73) | 0.004 |
|  |  |  |  |  |  |  |  |  |  |  |
| **FRACTURE** |  |  |  |  |  |  |  |  |  |  |
| No current use | 1658 | 349,837 | 1.00 |  |  | 1101 | 160,336 | 1.00 |  |  |
| TCAs | 109 | 20,204 | 1.10 | (0.90 to 1.34) | 0.37 | 96 | 17,105 | 0.76 | (0.61 to 0.94) | 0.012 |
| SSRIs | 812 | 130,683 | 1.36 | (1.24 to 1.49) | <0.001 | 654 | 73,909 | 1.25 | (1.13 to 1.38) | <0.001 |
| Other antidepressants | 122 | 15,331 | 1.50 | (1.25 to 1.80) | <0.001 | 73 | 9,834 | 1.04 | (0.82 to 1.32) | 0.73 |
| Combined antidepressants | 9 | 2,086 | 0.80 | (0.42 to 1.56) | 0.52 | 20 | 1,672 | 1.62 | (1.03 to 2.55) | 0.038 |
|  |  |  |  |  |  |  |  |  |  |  |
| **UPPER GI BLEED** |  |  |  |  |  |  |  |  |  |  |
| No current use | 362 | 391,625 | 1.00 |  |  | 226 | 179,029 | 1.00 |  |  |
| TCAs | 34 | 22,468 | 1.32 | (0.94 to 1.84) | 0.11 | 45 | 18,827 | 1.49 | (1.05 to 2.11) | 0.024 |
| SSRIs | 189 | 144,496 | 1.32 | (1.09 to 1.59) | 0.004 | 111 | 81,840 | 0.97 | (0.78 to 1.21) | 0.81 |
| Other antidepressants | 33 | 17,169 | 1.66 | (1.15 to 2.39) | 0.007 | 17 | 10,933 | 0.98 | (0.58 to 1.67) | 0.95 |
| Combined antidepressants | 8 | 2,342 | 2.89 | (1.44 to 5.81) | 0.003 | 4 | 1,903 | 1.40 | (0.51 to 3.82) | 0.51 |
|  |  |  |  |  |  |  |  |  |  |  |
| **ROAD TRAFFIC CRASH** |  |  |  |  |  |  |  |  |  |  |
| No current use | 1890 | 373,475 | 1.00 |  |  | 453 | 173,440 | 1.00 |  |  |
| TCAs | 114 | 21,531 | 1.03 | (0.83 to 1.29) | 0.76 | 40 | 18,398 | 0.79 | (0.56 to 1.13) | 0.20 |
| SSRIs | 704 | 138,656 | 1.02 | (0.93 to 1.12) | 0.72 | 225 | 79,482 | 1.06 | (0.89 to 1.26) | 0.49 |
| Other antidepressants | 85 | 16,521 | 1.02 | (0.81 to 1.28) | 0.89 | 35 | 10,700 | 1.27 | (0.91 to 1.78) | 0.16 |
| Combined antidepressants | 12 | 2,226 | 1.06 | (0.61 to 1.85) | 0.83 | 6 | 1,837 | 1.23 | (0.55 to 2.77) | 0.62 |
|  |  |  |  |  |  |  |  |  |  |  |
|  |  |  |  |  |  |  |  |  |  |  |
|  |  |  |  |  |  |  |  |  |  |  |
| **ADVERSE DRUG REACTION** |  |  |  |  |  |  |  |  |  |  |
| No current use | 243 | 392,177 | 1.00 |  |  | 189 | 179,224 | 1.00 |  |  |
| TCAs | 58 | 22,536 | 2.96 | (2.15 to 4.07) | <0.001 | 64 | 18,844 | 2.37 | (1.74 to 3.22) | <0.001 |
| SSRIs | 249 | 144,668 | 2.10 | (1.71 to 2.59) | <0.001 | 147 | 81,909 | 1.34 | (1.07 to 1.69) | 0.011 |
| Other antidepressants | 47 | 17,217 | 3.83 | (2.67 to 5.50) | <0.001 | 23 | 10,980 | 1.78 | (1.13 to 2.79) | 0.012 |
| Combined antidepressants | 8 | 2,350 | 4.92 | (2.44 to 9.92) | <0.001 | 3 | 1,907 | 1.36 | (0.43 to 4.29) | 0.60 |
|  |  |  |  |  |  |  |  |  |  |  |
| **ALL-CAUSE MORTALITY** |  |  |  |  |  |  |  |  |  |  |
| No current use | 460 | 394,758 | 1.00 |  |  | 1081 | 180,864 | 1.00 |  |  |
| TCAs | 55 | 22,743 | 1.80 | (1.35 to 2.41) | <0.001 | 271 | 19,064 | 1.96 | (1.68 to 2.28) | <0.001 |
| SSRIs | 206 | 145,580 | 1.29 | (1.07 to 1.55) | 0.007 | 784 | 82,654 | 1.43 | (1.30 to 1.58) | <0.001 |
| Other antidepressants | 43 | 17,378 | 1.62 | (1.19 to 2.23) | 0.003 | 153 | 11,109 | 1.81 | (1.50 to 2.18) | <0.001 |
| Combined antidepressants | 10 | 2,374 | 2.35 | (1.24 to 4.44) | 0.009 | 31 | 1,926 | 2.15 | (1.50 to 3.08) | <0.001 |
|  |  |  |  |  |  |  |  |  |  |  |

SSRIs=selective serotonin reuptake inhibitors; TCAs=tricyclic and related antidepressants.

* Based on numbers in adjusted analysis

† Adjusted for age, sex, year of diagnosis of depression, severity of depression, deprivation, smoking status, alcohol intake, ethnic group (white/not recorded or non-white), coronary heart disease, diabetes, hypertension, cancer, epilepsy/seizures, hypothyroidism, osteoarthritis, asthma/chronic obstructive airways disease, stroke/TIA, rheumatoid arthritis, osteoporosis, liver disease, renal disease, obsessive-compulsive disorder, statins, NSAIDS, aspirin, antihypertensive drugs, anticonvulsants, hypnotics/anxiolytics, oral contraceptives, hormone replacement therapy, antipsychotics, bisphosphonates, anticoagulants. Fracture outcome also adjusted for falls.

Table S3 Unadjusted and adjusted hazard ratios for 6 adverse outcomes (falls, fracture, upper gastrointestinal bleed, adverse drug reaction, road traffic crash, and all-cause mortality) by antidepressant drug, compared with citalopram over 1 year’s follow-up.

|  |  |  | Unadjusted analysis | | Adjusted analysis^1^ | | |
| --- | --- | --- | --- | --- | --- | --- | --- |
| **Antidepressant drug** | No of events^*^ | Person years^*^ | Hazard ratio | 95% CI | Hazard ratio | 95% CI | P |
|  |  |  |  |  |  |  |  |
| **FALLS** |  |  |  |  |  |  |  |
| *SSRIs:* |  |  |  |  |  |  |  |
| Citalopram | 223 | 40,355 | 1.00 |  | 1.00 |  |  |
| Escitalopram | 34 | 5,260 | 1.18 | (0.85 to 1.64) | 1.17 | (0.83 to 1.63) | 0.37 |
| Fluoxetine | 208 | 37,902 | 1.00 | (0.82 to 1.22) | 1.02 | (0.83 to 1.24) | 0.87 |
| Paroxetine | 44 | 7,800 | 1.00 | (0.72 to 1.39) | 1.08 | (0.77 to 1.51) | 0.67 |
| Sertraline | 38 | 7,515 | 0.89 | (0.62 to 1.27) | 0.92 | (0.65 to 1.31) | 0.65 |
| *TCAs:* |  |  |  |  |  |  |  |
| Amitriptyline | 49 | 6,805 | 1.42 | (1.04 to 1.92) | 1.05 | (0.78 to 1.43) | 0.74 |
| Dosulepin | 27 | 5,463 | 0.92 | (0.61 to 1.40) | 0.80 | (0.52 to 1.23) | 0.31 |
| Lofepramine | 7 | 2,274 | 0.54 | (0.26 to 1.14) | 0.54 | (0.26 to 1.12) | 0.10 |
| Trazodone | 3 | 828 | 0.85 | (0.34 to 2.12) | 0.58 | (0.20 to 1.68) | 0.32 |
| *Others:* |  |  |  |  |  |  |  |
| Mirtazapine | 22 | 3,211 | 1.29 | (0.86 to 1.95) | 1.11 | (0.71 to 1.71) | 0.65 |
| Venlafaxine | 18 | 4,293 | 0.76 | (0.48 to 1.19) | 0.77 | (0.48 to 1.23) | 0.28 |
|  |  |  |  |  |  |  |  |
| All other antidepressants | 5 | 1,607 | 0.66 | (0.29 to 1.50) | 0.51 | (0.21 to 1.24) | 0.14 |
| Combined antidepressants | 6 | 848 | 1.41 | (0.68 to 2.94) | 1.12 | (0.51 to 2.48) | 0.78 |
| No current use | 421 | 93,760 | 0.78 | (0.65 to 0.93) | 0.80 | (0.67 to 0.96) | 0.014 |
| Comparison between drugs^a^ |  |  |  |  |  |  | 0.63 |
|  |  |  |  |  |  |  |  |
| **FRACTURE** |  |  |  |  |  |  |  |
| *SSRIs:* |  |  |  |  |  |  |  |
| Citalopram | 255 | 36,936 | 1.00 |  | 1.00 |  |  |
| Escitalopram | 32 | 4,871 | 0.92 | (0.64 to 1.31) | 0.97 | (0.68 to 1.39) | 0.88 |
| Fluoxetine | 226 | 34,886 | 0.93 | (0.77 to 1.11) | 0.99 | (0.82 to 1.19) | 0.89 |
| Paroxetine | 49 | 7,228 | 0.97 | (0.71 to 1.31) | 1.07 | (0.77 to 1.47) | 0.70 |
| Sertraline | 46 | 6,901 | 0.99 | (0.72 to 1.36) | 1.00 | (0.72 to 1.39) | 1.00 |
| *TCAs:* |  |  |  |  |  |  |  |
| Amitriptyline | 33 | 6,265 | 0.79 | (0.55 to 1.11) | 0.72 | (0.50 to 1.03) | 0.07 |
| Dosulepin | 27 | 5,048 | 0.75 | (0.50 to 1.12) | 0.78 | (0.52 to 1.18) | 0.24 |
| Lofepramine | 8 | 2,089 | 0.59 | (0.32 to 1.10) | 0.57 | (0.29 to 1.13) | 0.11 |
| Trazodone | 3 | 748 | 0.74 | (0.27 to 2.02) | 0.51 | (0.16 to 1.59) | 0.24 |
| *Others:* |  |  |  |  |  |  |  |
| Mirtazapine | 22 | 2,866 | 1.10 | (0.72 to 1.69) | 0.91 | (0.58 to 1.41) | 0.67 |
| Venlafaxine | 33 | 3,924 | 1.16 | (0.81 to 1.65) | 1.22 | (0.85 to 1.75) | 0.29 |
|  |  |  |  |  |  |  |  |
| All other antidepressants | 11 | 1,477 | 1.04 | (0.57 to 1.91) | 1.06 | (0.58 to 1.94) | 0.84 |
| Combined antidepressants | 6 | 779 | 1.06 | (0.48 to 2.37) | 1.00 | (0.45 to 2.24) | 1.00 |
| No current use | 484 | 85,735 | 0.80 | (0.68 to 0.95) | 0.86 | (0.72 to 1.02) | 0.08 |
| Comparison between drugs^a^ |  |  |  |  |  |  | 0.32 |
|  |  |  |  |  |  |  |  |
| **UPPER GI BLEED** |  |  |  |  |  |  |  |
| *SSRIs:* |  |  |  |  |  |  |  |
| Citalopram | 53 | 40,957 | 1.00 |  | 1.00 |  |  |
| Escitalopram | 10 | 5,333 | 1.44 | (0.73 to 2.85) | 1.45 | (0.73 to 2.89) | 0.29 |
| Fluoxetine | 64 | 38,373 | 1.27 | (0.88 to 1.82) | 1.29 | (0.90 to 1.87) | 0.17 |
| Paroxetine | 14 | 7,878 | 1.37 | (0.76 to 2.45) | 1.28 | (0.71 to 2.32) | 0.41 |
| Sertraline | 4 | 7,604 | 0.51 | (0.20 to 1.26) | 0.40 | (0.14 to 1.10) | 0.08 |
| *TCAs:* |  |  |  |  |  |  |  |
| Amitriptyline | 15 | 6,930 | 1.65 | (0.94 to 2.90) | 1.39 | (0.78 to 2.47) | 0.26 |
| Dosulepin | 9 | 5,533 | 1.39 | (0.72 to 2.67) | 1.12 | (0.57 to 2.20) | 0.73 |
| Lofepramine | 4 | 2,299 | 1.31 | (0.48 to 3.56) | 1.19 | (0.44 to 3.23) | 0.73 |
| Trazodone | 4 | 826 | 3.70 | (1.32 to 10.34) | 2.87 | (1.01 to 8.12) | 0.047 |
| *Others:* |  |  |  |  |  |  |  |
| Mirtazapine | 8 | 3,237 | 2.15 | (1.05 to 4.39) | 1.39 | (0.66 to 2.97) | 0.39 |
| Venlafaxine | 19 | 4,320 | 3.64 | (2.23 to 5.94) | 3.10 | (1.88 to 5.11) | <0.001 |
|  |  |  |  |  |  |  |  |
| All other antidepressants | 4 | 1,631 | 2.36 | (0.95 to 5.86) | 1.72 | (0.62 to 4.80) | 0.30 |
| Combined antidepressants | 3 | 861 | 2.75 | (0.86 to 8.82) | 2.14 | (0.70 to 6.57) | 0.18 |
| No current use | 108 | 95,101 | 0.97 | (0.70 to 1.33) | 0.90 | (0.65 to 1.24) | 0.51 |
| Comparison between drugs^a^ |  |  |  |  |  |  | 0.002 |
|  |  |  |  |  |  |  |  |
| **ROAD TRAFFIC CRASH** |  |  |  |  |  |  |  |
| *SSRIs:* |  |  |  |  |  |  |  |
| Citalopram | 184 | 39,441 | 1.00 |  | 1.00 |  |  |
| Escitalopram | 29 | 5,150 | 1.25 | (0.84 to 1.86) | 1.23 | (0.81 to 1.86) | 0.32 |
| Fluoxetine | 148 | 37,113 | 0.88 | (0.71 to 1.09) | 0.85 | (0.68 to 1.07) | 0.16 |
| Paroxetine | 42 | 7,655 | 1.22 | (0.86 to 1.72) | 1.21 | (0.83 to 1.76) | 0.32 |
| Sertraline | 38 | 7,371 | 1.06 | (0.76 to 1.49) | 1.09 | (0.77 to 1.53) | 0.63 |
| *TCAs:* |  |  |  |  |  |  |  |
| Amitriptyline | 24 | 6,687 | 0.76 | (0.46 to 1.26) | 0.81 | (0.48 to 1.36) | 0.43 |
| Dosulepin | 13 | 5,378 | 0.55 | (0.33 to 0.91) | 0.58 | (0.34 to 0.99) | 0.047 |
| Lofepramine | 8 | 2,244 | 0.73 | (0.33 to 1.59) | 0.77 | (0.36 to 1.69) | 0.52 |
| Trazodone | 3 | 807 | 0.77 | (0.25 to 2.31) | 0.86 | (0.28 to 2.60) | 0.79 |
| *Others:* |  |  |  |  |  |  |  |
| Mirtazapine | 16 | 3,128 | 1.10 | (0.66 to 1.84) | 1.11 | (0.66 to 1.89) | 0.69 |
| Venlafaxine | 18 | 4,203 | 0.96 | (0.61 to 1.52) | 0.93 | (0.58 to 1.49) | 0.76 |
|  |  |  |  |  |  |  |  |
| All other antidepressants | 10 | 1,583 | 1.31 | (0.71 to 2.42) | 1.43 | (0.77 to 2.66) | 0.26 |
| Combined antidepressants | 3 | 832 | 0.73 | (0.24 to 2.26) | 0.77 | (0.25 to 2.39) | 0.65 |
| No current use | 415 | 91,721 | 0.96 | (0.81 to 1.13) | 0.92 | (0.77 to 1.09) | 0.33 |
| Comparison between drugs^a^ |  |  |  |  |  |  | 0.23 |
|  |  |  |  |  |  |  |  |
| **ADVERSE DRUG REACTION** |  |  |  |  |  |  |  |
| *SSRIs:* |  |  |  |  |  |  |  |
| Citalopram | 84 | 40,982 | 1.00 |  | 1.00 |  |  |
| Escitalopram | 14 | 5,327 | 1.25 | (0.72 to 2.18) | 1.29 | (0.73 to 2.25) | 0.38 |
| Fluoxetine | 78 | 38,432 | 1.00 | (0.74 to 1.36) | 0.98 | (0.72 to 1.35) | 0.92 |
| Paroxetine | 25 | 7,886 | 1.50 | (0.95 to 2.37) | 1.61 | (1.00 to 2.59) | 0.049 |
| Sertraline | 26 | 7,613 | 1.68 | (1.12 to 2.51) | 1.75 | (1.16 to 2.64) | 0.008 |
| *TCAs:* |  |  |  |  |  |  |  |
| Amitriptyline | 26 | 6,951 | 1.68 | (1.05 to 2.07) | 1.56 | (0.96 to 2.55) | 0.07 |
| Dosulepin | 16 | 5,537 | 1.33 | (0.77 to 2.29) | 1.34 | (0.76 to 2.34) | 0.31 |
| Lofepramine | 16 | 2,301 | 3.24 | (1.91 to 5.51) | 3.44 | (2.01 to 5.88) | <0.001 |
| Trazodone | 0 | 833 | - | - | - | - | - |
| *Others:* |  |  |  |  |  |  |  |
| Mirtazapine | 10 | 3,254 | 1.78 | (0.90 to 3.52) | 1.67 | (0.82 to 3.39) | 0.16 |
| Venlafaxine | 17 | 4,334 | 2.21 | (1.17 to 4.19) | 2.34 | (1.24 to 4.43) | 0.009 |
|  |  |  |  |  |  |  |  |
| All other antidepressants | 7 | 1,635 | 2.00 | (0.97 to 4.13) | 2.00 | (0.95 to 4.2) | 0.07 |
| Combined antidepressants | 4 | 861 | 2.70 | (0.98 to 7.43) | 2.80 | (1.01 to 7.75) | 0.047 |
| No current use | 91 | 95,252 | 0.58 | (0.44 to 0.76) | 0.62 | (0.46 to 0.82) | 0.001 |
| Comparison between drugs^a^ |  |  |  |  |  |  | <0.001 |
|  |  |  |  |  |  |  |  |
| **ALL-CAUSE MORTALITY** |  |  |  |  |  |  |  |
| *SSRIs:* |  |  |  |  |  |  |  |
| Citalopram | 173 | 41,234 | 1.00 |  | 1.00 |  |  |
| Escitalopram | 24 | 5,363 | 1.09 | (0.73 to 1.62) | 1.05 | (0.70 to 1.59) | 0.81 |
| Fluoxetine | 147 | 38,637 | 0.90 | (0.73 to 1.12) | 0.97 | (0.77 to 1.21) | 0.76 |
| Paroxetine | 24 | 7,928 | 0.76 | (0.51 to 1.15) | 0.79 | (0.51 to 1.24) | 0.31 |
| Sertraline | 33 | 7,660 | 1.04 | (0.72 to 1.49) | 1.04 | (0.72 to 1.49) | 0.84 |
| *TCAs:* |  |  |  |  |  |  |  |
| Amitriptyline | 52 | 7,006 | 1.72 | (1.27 to 2.33) | 1.36 | (0.99 to 1.86) | 0.06 |
| Dosulepin | 17 | 5,573 | 0.75 | (0.45 to 1.27) | 0.60 | (0.35 to 1.05) | 0.07 |
| Lofepramine | 14 | 2,318 | 1.47 | (0.87 to 2.51) | 1.25 | (0.69 to 2.25) | 0.46 |
| Trazodone | 3 | 837 | 0.83 | (0.27 to 2.59) | 0.64 | (0.21 to 1.93) | 0.43 |
| *Others:* |  |  |  |  |  |  |  |
| Mirtazapine | 34 | 3,285 | 2.49 | (1.72 to 3.60) | 1.63 | (1.12 to 2.38) | 0.011 |
| Venlafaxine | 18 | 4,360 | 0.98 | (0.61 to 1.57) | 0.94 | (0.58 to 1.51) | 0.78 |
|  |  |  |  |  |  |  |  |
| All other antidepressants | 13 | 1,647 | 1.98 | (1.16 to 3.37) | 1.43 | (0.83 to 2.48) | 0.20 |
| Combined antidepressants | 8 | 868 | 2.20 | (1.08 to 4.46) | 1.66 | (0.79 to 3.48) | 0.18 |
| No current use | 298 | 95,801 | 0.79 | (0.66 to 0.96) | 0.91 | (0.75 to 1.10) | 0.33 |
| Comparison between drugs^a^ |  |  |  |  |  |  | 0.036 |
|  |  |  |  |  |  |  |  |

* Based on numbers in adjusted analysis

SSRIs=selective serotonin reuptake inhibitors; TCAs=tricyclic and related antidepressants.

^1^ Adjusted for age, sex, year of diagnosis of depression, severity of depression, deprivation, smoking status, alcohol intake, ethnic group (white/not recorded or non-white), coronary heart disease, diabetes, hypertension, cancer, epilepsy/seizures, hypothyroidism, osteoarthritis, asthma/chronic obstructive airways disease, stroke/TIA, rheumatoid arthritis, osteoporosis, liver disease, renal disease, obsessive-compulsive disorder, statins, NSAIDS, aspirin, antihypertensive drugs, anticonvulsants, hypnotics/anxiolytics, oral contraceptives, hormone replacement therapy, antipsychotics, bisphosphonates, anticoagulants. Fracture outcome also adjusted for falls.

^a^ Comparison is between the 11 individual drugs (Wald’s test)

Table S4 Adjusted hazard ratios for six adverse outcomes (falls, fracture, upper gastrointestinal bleed, adverse drug reaction, road traffic crash, and all-cause mortality) by antidepressant class compared with no use of antidepressants according to duration of use and time since stopping for each antidepressant class over 5 years follow-up

|  | **No of events^*^** | **Person years^*^** | **Adjusted hazard ratio†** | **95% CI** | **P value** |
| --- | --- | --- | --- | --- | --- |
|  |  |  |  |  |  |
| **FALLS** |  |  |  |  |  |
| No current or recent use | 2212 | 501,800 | 1.00 |  |  |
| *TCAs:* |  |  |  |  |  |
| first 28 days | 26 | 5,360 | 0.99 | (0.66 to 1.49) | 0.96 |
| 29 to 84 days after starting | 47 | 5,282 | 1.82 | (1.37 to 2.42) | <0.001 |
| 85 or more days after starting | 151 | 18,485 | 1.36 | (1.14 to 1.63) | 0.001 |
| 1-28 days after stopping | 18 | 3,535 | 1.07 | (0.66 to 1.72) | 0.80 |
| 29-84 days after stopping | 54 | 6,875 | 1.61 | (1.22 to 2.14) | 0.001 |
| 85-182 days after stopping | 66 | 10,475 | 1.27 | (1.01 to 1.61) | 0.045 |
| *SSRIs:* |  |  |  |  |  |
| first 28 days | 104 | 20,364 | 1.28 | (1.03 to 1.60) | 0.028 |
| 29 to 84 days after starting | 165 | 27,514 | 1.52 | (1.28 to 1.81) | <0.001 |
| 85 or more days after starting | 948 | 125,402 | 1.56 | (1.44 to 1.68) | <0.001 |
| 1-28 days after stopping | 94 | 15,449 | 1.53 | (1.24 to 1.88) | <0.001 |
| 29-84 days after stopping | 168 | 29,937 | 1.39 | (1.18 to 1.63) | <0.001 |
| 85-182 days after stopping | 203 | 46,079 | 1.06 | (0.92 to 1.23) | 0.41 |
| *Others:* |  |  |  |  |  |
| first 28 days | 19 | 2,739 | 1.60 | (1.02 to 2.50) | 0.041 |
| 29 to 84 days after starting | 23 | 3,457 | 1.53 | (1.03 to 2.28) | 0.034 |
| 85 or more days after starting | 126 | 16,630 | 1.53 | (1.27 to 1.85) | <0.001 |
| 1-28 days after stopping | 8 | 1,553 | 1.21 | (0.60 to 2.40) | 0.60 |
| 29-84 days after stopping | 11 | 2,985 | 0.85 | (0.45 to 1.61) | 0.62 |
| 85-182 days after stopping | 30 | 4,480 | 1.53 | (1.09 to 2.16) | 0.014 |
|  |  |  |  |  |  |
| **FRACTURE** |  |  |  |  |  |
| No current or recent use | 2465 | 457,780 | 1.00 |  |  |
| *TCAs:* |  |  |  |  |  |
| first 28 days | 30 | 4,943 | 1.17 | (0.83 to 1.65) | 0.38 |
| 29 to 84 days after starting | 36 | 4,878 | 1.26 | (0.90 to 1.75) | 0.18 |
| 85 or more days after starting | 83 | 17,206 | 0.77 | (0.61 to 0.98) | 0.030 |
| 1-28 days after stopping | 21 | 3,265 | 1.12 | (0.74 to 1.70) | 0.59 |
| 29-84 days after stopping | 28 | 6,349 | 0.77 | (0.53 to 1.12) | 0.17 |
| 85-182 days after stopping | 57 | 9,666 | 1.05 | (0.80 to 1.37) | 0.74 |
| *SSRIs:* |  |  |  |  |  |
| first 28 days | 106 | 18,724 | 1.25 | (0.99 to 1.57) | 0.06 |
| 29 to 84 days after starting | 201 | 25,314 | 1.48 | (1.26 to 1.73) | <0.001 |
| 85 or more days after starting | 879 | 115,981 | 1.33 | (1.23 to 1.45) | <0.001 |
| 1-28 days after stopping | 94 | 14,192 | 1.23 | (1.00 to 1.53) | 0.05 |
| 29-84 days after stopping | 168 | 27,492 | 1.15 | (0.98 to 1.34) | 0.08 |
| 85-182 days after stopping | 238 | 42,292 | 1.07 | (0.93 to 1.22) | 0.37 |
| *Others:* |  |  |  |  |  |
| first 28 days | 18 | 2,481 | 1.29 | (0.82 to 2.04) | 0.26 |
| 29 to 84 days after starting | 38 | 3,135 | 2.03 | (1.47 to 2.82) | <0.001 |
| 85 or more days after starting | 113 | 15,200 | 1.21 | (1.00 to 1.46) | 0.048 |
| 1-28 days after stopping | 10 | 1,393 | 1.23 | (0.66 to 2.28) | 0.52 |
| 29-84 days after stopping | 16 | 2,675 | 1.03 | (0.63 to 1.68) | 0.92 |
| 85-182 days after stopping | 23 | 4,015 | 0.99 | (0.64 to 1.54) | 0.97 |
|  |  |  |  |  |  |
| **UPPER GI BLEED** |  |  |  |  |  |
| No current or recent use | 512 | 512,430 | 1.00 |  |  |
| *TCAs:* |  |  |  |  |  |
| first 28 days | 13 | 5,488 | 1.79 | (0.97 to 3.29) | 0.06 |
| 29 to 84 days after starting | 14 | 5,404 | 2.11 | (1.26 to 3.55) | 0.005 |
| 85 or more days after starting | 36 | 19,003 | 1.46 | (1.03 to 2.06) | 0.032 |
| 1-28 days after stopping | 8 | 3,619 | 1.86 | (0.84 to 4.09) | 0.13 |
| 29-84 days after stopping | 8 | 7,040 | 0.92 | (0.45 to 1.85) | 0.81 |
| 85-182 days after stopping | 22 | 10,724 | 1.68 | (1.09 to 2.59) | 0.020 |
| *SSRIs:* |  |  |  |  |  |
| first 28 days | 34 | 20,705 | 1.37 | (0.87 to 2.15) | 0.17 |
| 29 to 84 days after starting | 44 | 27,970 | 1.53 | (1.06 to 2.21) | 0.022 |
| 85 or more days after starting | 151 | 128,248 | 1.07 | (0.89 to 1.30) | 0.46 |
| 1-28 days after stopping | 30 | 15,726 | 1.82 | (1.26 to 2.63) | 0.002 |
| 29-84 days after stopping | 38 | 30,483 | 1.15 | (0.82 to 1.60) | 0.42 |
| 85-182 days after stopping | 49 | 46,942 | 0.98 | (0.72 to 1.33) | 0.90 |
| *Others:* |  |  |  |  |  |
| first 28 days | 10 | 2,777 | 2.68 | (1.43 to 5.04) | 0.002 |
| 29 to 84 days after starting | 7 | 3,504 | 1.55 | (0.74 to 3.27) | 0.25 |
| 85 or more days after starting | 27 | 16,914 | 1.26 | (0.87 to 1.83) | 0.22 |
| 1-28 days after stopping | 3 | 1,571 | 1.52 | (0.48 to 4.77) | 0.48 |
| 29-84 days after stopping | 3 | 3,020 | 0.78 | (0.25 to 2.41) | 0.66 |
| 85-182 days after stopping | 8 | 4,532 | 1.41 | (0.71 to 2.81) | 0.32 |
|  |  |  |  |  |  |
| **ROAD TRAFFIC CRASH** |  |  |  |  |  |
| No current or recent use | 2060 | 490,916 | 1.00 |  |  |
| *TCAs:* |  |  |  |  |  |
| first 28 days | 26 | 5,288 | 1.25 | (0.83 to 1.89) | 0.29 |
| 29 to 84 days after starting | 23 | 5,218 | 1.16 | (0.76 to 1.78) | 0.49 |
| 85 or more days after starting | 62 | 18,452 | 0.88 | (0.68 to 1.16) | 0.37 |
| 1-28 days after stopping | 13 | 3,484 | 0.95 | (0.54 to 1.69) | 0.87 |
| 29-84 days after stopping | 30 | 6,775 | 1.06 | (0.73 to 1.53) | 0.77 |
| 85-182 days after stopping | 53 | 10,321 | 1.20 | (0.90 to 1.59) | 0.21 |
| *SSRIs:* |  |  |  |  |  |
| first 28 days | 74 | 19,961 | 0.92 | (0.71 to 1.20) | 0.56 |
| 29 to 84 days after starting | 108 | 26,959 | 1.05 | (0.84 to 1.30) | 0.67 |
| 85 or more days after starting | 502 | 123,663 | 1.02 | (0.92 to 1.14) | 0.67 |
| 1-28 days after stopping | 72 | 15,140 | 1.15 | (0.91 to 1.46) | 0.24 |
| 29-84 days after stopping | 158 | 29,332 | 1.22 | (1.03 to 1.45) | 0.022 |
| 85-182 days after stopping | 227 | 45,135 | 1.12 | (0.97 to 1.30) | 0.12 |
| *Others:* |  |  |  |  |  |
| first 28 days | 17 | 2,682 | 1.57 | (0.98 to 2.53) | 0.06 |
| 29 to 84 days after starting | 17 | 3,388 | 1.25 | (0.78 to 1.99) | 0.36 |
| 85 or more days after starting | 63 | 16,414 | 0.98 | (0.76 to 1.27) | 0.88 |
| 1-28 days after stopping | 5 | 1,517 | 0.80 | (0.33 to 1.92) | 0.62 |
| 29-84 days after stopping | 17 | 2,914 | 1.36 | (0.87 to 2.14) | 0.18 |
| 85-182 days after stopping | 18 | 4,368 | 0.96 | (0.59 to 1.53) | 0.85 |
|  |  |  |  |  |  |
|  |  |  |  |  |  |
| **ADVERSE DRUG REACTION** |  |  |  |  |  |
| No current or recent use | 380 | 513,095 | 1.00 |  |  |
| *TCAs:* |  |  |  |  |  |
| first 28 days | 53 | 5,500 | 8.72 | (6.25 to 12.16) | <0.001 |
| 29 to 84 days after starting | 15 | 5,416 | 2.74 | (1.55 to 4.84) | 0.001 |
| 85 or more days after starting | 35 | 19,048 | 2.01 | (1.41 to 2.86) | <0.001 |
| 1-28 days after stopping | 8 | 3,625 | 2.32 | (1.15 to 4.67) | 0.018 |
| 29-84 days after stopping | 11 | 7,050 | 1.82 | (0.96 to 3.46) | 0.07 |
| 85-182 days after stopping | 14 | 10,739 | 1.53 | (0.90 to 2.62) | 0.12 |
| *SSRIs:* |  |  |  |  |  |
| first 28 days | 143 | 20,737 | 6.27 | (4.74 to 8.28) | <0.001 |
| 29 to 84 days after starting | 54 | 28,000 | 2.01 | (1.42 to 2.84) | <0.001 |
| 85 or more days after starting | 145 | 128,365 | 1.37 | (1.12 to 1.67) | 0.002 |
| 1-28 days after stopping | 25 | 15,745 | 1.85 | (1.23 to 2.78) | 0.003 |
| 29-84 days after stopping | 26 | 30,520 | 1.09 | (0.71 to 1.67) | 0.70 |
| 85-182 days after stopping | 37 | 46,994 | 1.02 | (0.71 to 1.46) | 0.94 |
| *Others:* |  |  |  |  |  |
| first 28 days | 33 | 2,786 | 12.37 | (8.21 to 18.63) | <0.001 |
| 29 to 84 days after starting | 12 | 3,512 | 3.90 | (2.23 to 6.82) | <0.001 |
| 85 or more days after starting | 21 | 16,971 | 1.53 | (0.96 to 2.42) | 0.07 |
| 1-28 days after stopping | 2 | 1,578 | 1.52 | (0.38 to 6.11) | 0.56 |
| 29-84 days after stopping | 2 | 3,032 | 0.84 | (0.21 to 3.34) | 0.80 |
| 85-182 days after stopping | 4 | 4,554 | 1.13 | (0.44 to 2.93) | 0.80 |
|  |  |  |  |  |  |
| **MORTALITY** |  |  |  |  |  |
| No current or recent use | 1284 | 516,883 | 1.00 |  |  |
| *TCAs:* |  |  |  |  |  |
| first 28 days | 35 | 5,551 | 2.11 | (1.47 to 3.02) | <0.001 |
| 29 to 84 days after starting | 24 | 5,467 | 1.38 | (0.91 to 2.09) | 0.13 |
| 85 or more days after starting | 73 | 19,267 | 0.89 | (0.68 to 1.16) | 0.38 |
| 1-28 days after stopping | 101 | 3,658 | 9.53 | (7.62 to 11.92) | <0.001 |
| 29-84 days after stopping | 87 | 7,115 | 4.32 | (3.43 to 5.44) | <0.001 |
| 85-182 days after stopping | 62 | 10,840 | 2.03 | (1.55 to 2.66) | <0.001 |
| *SSRIs:* |  |  |  |  |  |
| first 28 days | 95 | 20,872 | 2.16 | (1.69 to 2.77) | <0.001 |
| 29 to 84 days after starting | 67 | 28,185 | 1.06 | (0.81 to 1.37) | 0.68 |
| 85 or more days after starting | 297 | 129,369 | 0.74 | (0.64 to 0.85) | <0.001 |
| 1-28 days after stopping | 281 | 15,850 | 8.40 | (7.30 to 9.66) | <0.001 |
| 29-84 days after stopping | 233 | 30,726 | 3.63 | (3.10 to 4.25) | <0.001 |
| 85-182 days after stopping | 180 | 47,314 | 1.80 | (1.53 to 2.13) | <0.001 |
| *Others:* |  |  |  |  |  |
| first 28 days | 25 | 2,814 | 2.94 | (1.96 to 4.41) | <0.001 |
| 29 to 84 days after starting | 12 | 3,548 | 1.07 | (0.60 to 1.90) | 0.82 |
| 85 or more days after starting | 58 | 17,150 | 0.86 | (0.65 to 1.14) | 0.31 |
| 1-28 days after stopping | 63 | 1,593 | 13.35 | (10.37 to 17.18) | <0.001 |
| 29-84 days after stopping | 34 | 3,060 | 3.80 | (2.71 to 5.33) | <0.001 |
| 85-182 days after stopping | 36 | 4,594 | 2.70 | (1.91 to 3.82) | <0.001 |
|  |  |  |  |  |  |

SSRIs=selective serotonin reuptake inhibitors; TCAs=tricyclic and related antidepressants.

* Based on numbers in adjusted analysis

† Adjusted for age, sex, year of diagnosis of depression, severity of depression, deprivation, smoking status, alcohol intake, ethnic group (white/not recorded or non-white), coronary heart disease, diabetes, hypertension, cancer, epilepsy/seizures, hypothyroidism, osteoarthritis, asthma/chronic obstructive airways disease, stroke/TIA, rheumatoid arthritis, osteoporosis, liver disease, renal disease, obsessive-compulsive disorder, statins, NSAIDS, aspirin, antihypertensive drugs, anticonvulsants, hypnotics/anxiolytics, oral contraceptives, hormone replacement therapy, antipsychotics, bisphosphonates, anticoagulants. Fracture outcome also adjusted for falls.

**Table S5** Adjusted hazard ratios for six adverse outcomes (falls, fracture, upper gastrointestinal bleed, adverse drug reaction, road traffic crash, and all-cause mortality) by antidepressant class compared with no use of antidepressants, over (A) total follow-up time and (B) 5 years follow-up excluding untreated patients.

|  | 1. Total follow-up time | | | | | 1. 5 years follow-up excluding untreated patients | | | | |
| --- | --- | --- | --- | --- | --- | --- | --- | --- | --- | --- |
| **Antidepressant class** | No of events^*^ | Person years^*^ | Adjusted hazard ratio† | 95% CI | p | No of events^*^ | Person years^*^ | Adjusted hazard ratio† | 95% CI | P |
| **FALLS** |  |  |  |  |  |  |  |  |  |  |
| No current use | 3859 | 839,530 | 1.00 |  |  | 2167 | 463,879 | 1.00 |  |  |
| TCAs | 447 | 56,401 | 1.36 | (1.23 to 1.51) | <0.001 | 298 | 40,260 | 1.30 | (1.14 to 1.49) | <0.001 |
| SSRIs | 2024 | 283,178 | 1.52 | (1.43 to 1.61) | <0.001 | 1494 | 221,813 | 1.42 | (1.33 to 1.53) | <0.001 |
| Other antidepressants | 294 | 39,042 | 1.51 | (1.34 to 1.70) | <0.001 | 189 | 27,678 | 1.37 | (1.18 to 1.59) | <0.001 |
| Combined antidepressants | 80 | 7,022 | 1.91 | (1.53 to 2.39) | <0.001 | 37 | 4,121 | 1.54 | (1.12 to 2.13) | 0.009 |
|  |  |  |  |  |  |  |  |  |  |  |
| **FRACTURE** |  |  |  |  |  |  |  |  |  |  |
| No current use | 4117 | 765,678 | 1.00 |  |  | 2310 | 423,877 | 1.00 |  |  |
| TCAs | 322 | 52,558 | 1.03 | (0.92 to 1.16) | 0.59 | 205 | 37,309 | 0.92 | (0.80 to 1.07) | 0.30 |
| SSRIs | 1955 | 261,850 | 1.37 | (1.29 to 1.45) | <0.001 | 1466 | 204,592 | 1.30 | (1.21 to 1.40) | <0.001 |
| Other antidepressants | 277 | 35,637 | 1.31 | (1.16 to 1.48) | <0.001 | 195 | 25,164 | 1.28 | (1.11 to 1.48) | 0.001 |
| Combined antidepressants | 61 | 6,501 | 1.52 | (1.18 to 1.95) | 0.001 | 29 | 3,759 | 1.21 | (0.82 to 1.80) | 0.34 |
|  |  |  |  |  |  |  |  |  |  |  |
| **UPPER GI BLEED** |  |  |  |  |  |  |  |  |  |  |
| No current use | 839 | 862,219 | 1.00 |  |  | 498 | 473,818 | 1.00 |  |  |
| TCAs | 107 | 58,525 | 1.48 | (1.20 to 1.83) | <0.001 | 79 | 41,295 | 1.44 | (1.12 to 1.83) | 0.004 |
| SSRIs | 381 | 291,050 | 1.21 | (1.07 to 1.37) | 0.003 | 300 | 226,336 | 1.15 | (0.99 to 1.34) | 0.07 |
| Other antidepressants | 79 | 40,059 | 1.57 | (1.23 to 2.02) | <0.001 | 50 | 28,102 | 1.34 | (0.99 to 1.81) | 0.06 |
| Combined antidepressants | 17 | 7,370 | 1.86 | (1.14 to 3.02) | 0.012 | 12 | 4,245 | 2.10 | (1.17 to 3.76) | 0.013 |
|  |  |  |  |  |  |  |  |  |  |  |
| **ROAD TRAFFIC CRASH** |  |  |  |  |  |  |  |  |  |  |
| No current use | 3324 | 823,446 | 1.00 |  |  | 1978 | 453,993 | 1.00 |  |  |
| TCAs | 205 | 56,463 | 0.96 | (0.82 to 1.13) | 0.64 | 154 | 39,929 | 0.95 | (0.78 to 1.15) | 0.57 |
| SSRIs | 1140 | 279,619 | 1.03 | (0.95 to 1.11) | 0.46 | 929 | 218,137 | 1.01 | (0.93 to 1.10) | 0.82 |
| Other antidepressants | 157 | 38,692 | 1.05 | (0.89 to 1.23) | 0.56 | 120 | 27,221 | 1.06 | (0.88 to 1.28) | 0.52 |
| Combined antidepressants | 33 | 7,046 | 1.28 | (0.90 to 1.81) | 0.17 | 18 | 4,063 | 1.09 | (0.69 to 1.72) | 0.70 |
|  |  |  |  |  |  |  |  |  |  |  |
|  |  |  |  |  |  |  |  |  |  |  |
|  |  |  |  |  |  |  |  |  |  |  |
| **ADVERSE DRUG REACTION** |  |  |  |  |  |  |  |  |  |  |
| No current use | 674 | 863,228 | 1.00 |  |  | 358 | 474,469 | 1.00 |  |  |
| TCAs | 162 | 58,618 | 2.56 | (2.11 to 3.11) | <0.001 | 122 | 41,380 | 2.64 | (2.09 to 3.33) | <0.001 |
| SSRIs | 480 | 291,216 | 1.65 | (1.44 to 1.90) | <0.001 | 396 | 226,577 | 1.70 | (1.43 to 2.01) | <0.001 |
| Other antidepressants | 87 | 40,184 | 2.44 | (1.89 to 3.14) | <0.001 | 70 | 28,197 | 2.76 | (2.06 to 3.69) | <0.001 |
| Combined antidepressants | 14 | 7,411 | 2.09 | (1.24 to 3.54) | 0.006 | 11 | 4,257 | 2.90 | (1.58 to 5.34) | 0.001 |
|  |  |  |  |  |  |  |  |  |  |  |
| **ALL-CAUSE MORTALITY** |  |  |  |  |  |  |  |  |  |  |
| No current use | 2386 | 870,780 | 1.00 |  |  | 1223 | 478,057 | 1.00 |  |  |
| TCAs | 475 | 59,410 | 1.99 | (1.77 to 2.25) | <0.001 | 326 | 41,807 | 2.08 | (1.82 to 2.38) | <0.001 |
| SSRIs | 1368 | 293,742 | 1.53 | (1.41 to 1.65) | <0.001 | 990 | 228,233 | 1.49 | (1.35 to 1.64) | <0.001 |
| Other antidepressants | 284 | 40,667 | 1.82 | (1.58 to 2.08) | <0.001 | 196 | 28,487 | 1.85 | (1.57 to 2.19) | <0.001 |
| Combined antidepressants | 79 | 7,507 | 2.50 | (1.94 to 3.22) | <0.001 | 41 | 4,299 | 2.30 | (1.68 to 3.16) | <0.001 |
|  |  |  |  |  |  |  |  |  |  |  |

* Based on numbers in adjusted analysis

SSRIs=selective serotonin reuptake inhibitors; TCAs=tricyclic and related antidepressants.

^†^ Adjusted for age, sex, year of diagnosis of depression, severity of depression, deprivation, smoking status, alcohol intake, ethnic group (white/not recorded or non-white), coronary heart disease, diabetes, hypertension, cancer, epilepsy/seizures, hypothyroidism, osteoarthritis, asthma/chronic obstructive airways disease, stroke/TIA, rheumatoid arthritis, osteoporosis, liver disease, renal disease, obsessive-compulsive disorder, statins, NSAIDS, aspirin, antihypertensive drugs, anticonvulsants, hypnotics/anxiolytics, oral contraceptives, hormone replacement therapy, antipsychotics, bisphosphonates, anticoagulants. Fracture outcome also adjusted for falls.
